# Development of risk models of incident hypertension using machine learning on the HUNT study data

**DOI:** 10.1038/s41598-024-56170-7

**Published:** 2024-03-07

**Authors:** Filip Emil Schjerven, Emma Maria Lovisa Ingeström, Ingelin Steinsland, Frank Lindseth

**Affiliations:** 1 Department of Computer Science, Norwegian University of Science and Technology, Trondheim, Norway; 2 Department of Circulation and Medical Imaging, Norwegian University of Science and Technology, Trondheim, Norway; 3 Department of Mathematical Sciences, Norwegian University of Science and Technology, Trondheim, Norway

**Keywords:** Medical research, Hypertension, Mathematics and computing, Computer science, Statistics, Risk factors

## Abstract

In this study, we aimed to create an 11-year hypertension risk prediction model using data from the Trøndelag Health (HUNT) Study in Norway, involving 17 852 individuals (20–85 years; 38% male; 24% incidence rate) with blood pressure (BP) below the hypertension threshold at baseline (1995–1997). We assessed 18 clinical, behavioral, and socioeconomic features, employing machine learning models such as eXtreme Gradient Boosting (XGBoost), Elastic regression, K-Nearest Neighbor, Support Vector Machines (SVM) and Random Forest. For comparison, we used logistic regression and a decision rule as reference models and validated six external models, with focus on the Framingham risk model. The top-performing models consistently included XGBoost, Elastic regression and SVM. These models efficiently identified hypertension risk, even among individuals with optimal baseline BP (< 120/80 mmHg), although improvement over reference models was modest. The recalibrated Framingham risk model outperformed the reference models, approaching the best-performing ML models. Important features included age, systolic and diastolic BP, body mass index, height, and family history of hypertension. In conclusion, our study demonstrated that linear effects sufficed for a well-performing model. The best models efficiently predicted hypertension risk, even among those with optimal or normal baseline BP, using few features. The recalibrated Framingham risk model proved effective in our cohort.

## Introduction

Hypertension, a medical condition of persistent elevated blood pressure (BP), is estimated to indirectly contribute to around 10 million annual deaths worldwide and up to 10% of the world’s total health resource expenditure^1–3^. Current practices for determining interventions in hypertension management rely on BP measurements, age, and risk profiles of other diseases where hypertension is a major risk-factor^1^. Lifestyle changes are a key intervention component for all stages of hypertension and are effective in preventing and delaying the onset of hypertension^1,2^. A risk model allowing detection of individuals that are currently free from, but at the risk of developing, hypertension could be used to initiate personalized prevention strategies earlier.

Several models that mathematically combine clinical, behavioral, genetic and socioeconomic risk factors to predict risk have been proposed for incident hypertension^4–7^. Generally, performance has been reported by discrimination measures, i.e., measures quantifying a model’s ability to distinguish between individuals who develop the disease or not^8^. The discrimination performance vary considerably between studies developing risk models^7^. Yet, studies rarely included reference models to contextualize their results, which makes it difficult to objectively assess any improvement upon simpler alternatives. Further, few models have been externally validated. Of these, most have been validated once or twice, except for the Framingham risk model^9^, which has been validated more than 15 times in external studies^7^.

Multiple studies have applied machine learning (ML) to develop risk models for hypertension. Most of these used cross-sectional data, i.e., data collected from a single point in time, to develop models for identifying existing hypertension^7^. Fewer have used prospective data, i.e., data collected from the same individuals at two separate points in time, to develop risk models using ML^7,10–18^.

On model performance, discrimination was often higher in studies applying ML compared to those applying more traditional regression-based models^7^. Nevertheless, when ML models were directly compared to simpler models such as the logistic or Cox regression model, the net improvement in discrimination varied^10–15,19^. Other relevant performance measures such as calibration, i.e., measures on the agreement of predictions with observed outcomes, are often neglected in studies applying ML^7,8,10–18^.

In this study, our primary objective was to develop a risk model for incident hypertension and assess the potential of ML on model performance. Secondary objectives were to identify the features most important for obtaining well-performing models, and externally validate existing hypertension risk models.

## Materials and methods

The Transparent Reporting of a multivariable prediction model for Individual Prognosis Or Diagnosis (TRIPOD)^20^ checklist for this study is supplied as Supplementary Item S1.

### Data

A dataset was derived from the Trøndelag Health (HUNT) Study, originating from the now former county of Nord-Trøndelag in Norway. The HUNT Study constitutes a large population database for medical and health-related research including four health surveys over four decades^21^. In this study, baseline data was collected from HUNT2 (1995–1997, 69.5% participation rate) with endpoint derived from the follow-up in HUNT3 (2006–2008). Although data from HUNT1 (1984–1986) exists, we selected data from the HUNT2-HUNT3 cohort as it represents a more concurrent population (e.g., prevalence and treatment of hypertension). Data from the more recent HUNT4 (2017–2019) was not yet available at the initiation of this work. The study population derived from HUNT2-HUNT3 is ethnically homogenous of European descent (> 97% Caucasian), and socioeconomically comparable with other Northern European countries^22,23^. We included records from individuals participating in the HUNT2 and HUNT3 surveys:

- With complete information on BP measurements and use of BP medication, at both baseline and follow-up,
- without missing information on diabetes or cardiovascular disease (CVD) at baseline,
- with a BP below the hypertension threshold, not using BP medication, and free from both CVD and diabetes at baseline.

All individuals were ≥ 20 years of age. BP was measured using an automatic oscillometric device (Critikon Dinamap 845XT or 8100, GE Healthcare, Chicago, US; Dinamap XL 9301, Johnson & Johnson Medical Inc., New Brunswick US). Measurements were taken in the sitting position after 2 min of rest by trained personnel using standardized protocols. A total of three consecutive measurements were taken 1 min apart. The first measurement was used to calibrate the device, and the mean of the subsequent two recorded as BP^22,23^. Hypertension status was determined according to the European Society of Cardiology (ESC) and the European Society of Hypertension (ESH) guidelines, i.e., a systolic pressure of 140 mmHg or more, diastolic pressure of 90 mmHg or more, and/or current use of BP medication^1^. The process of applying exclusion criteria and a general data-flow diagram is shown in Supplementary Fig. S1. From the records of 65 003 participants at HUNT2, 35 626 met the inclusion criteria at baseline. Of these, 12 687 were lost to follow-up, leaving 22 939 records. We excluded a further 5 087 records due to missing feature data, leaving 17 852 records in a complete dataset used for analysis.

The features used in our study are well-established risk factors of both hypertension and CVD and are commonly used in risk modelling of incident hypertension^1,6^. We estimated physical activity using a novel metric, Personal Activity Intelligence (PAI). The PAI algorithm converts self-reported leisure time physical activity to an average weekly PAI score representative for the last year^24–27^. The HUNT Study protocol have been described in detail by Åsvold et al.^21^ and more information about how the features and outcome were collected can be found in Supplementary Table S1 and at https://hunt-db.medisin.ntnu.no/hunt-db/#/. All participants provided written informed consent and this study was pre-approved by the Regional Committee on Medical and Health Research Ethics of Norway (REK; 22,902; 2018/1824), and all methods were performed in accordance with the relevant guidelines and regulations.

### Data statistics

The complete dataset was stratified on outcome status, i.e., above or below hypertension threshold at follow-up, and described by summary statistics. We applied Welch t-tests or chi-square tests as appropriate to detail significant differences between those whose BP remained below the threshold and those who developed hypertension. The same tests were applied on applicable groups whenever summarized feature data was compared in subsequent analyses. Whenever multiple comparisons were performed, we applied Holm’s step-down correction^28^ to determine significance, using α = 0.05 on the m = 19 data dimensions, i.e., 18 features and one outcome.

### Preprocessing

As part of the model development and evaluation, the data was preprocessed by standardizing the numerical features. Further, categorical features were left as they were for the tree-based methods, and binary encoded for the remaining models. The parameters needed for standardization were estimated using only the training set to avoid the possibility of data leakage, i.e., inadvertently using information from the test set to develop the models.

### Modelling

To construct the risk model, we considered several ML modelling methods. Using all features, we included the following methods: eXtreme Gradient Boosting algorithm (XGBoost)^29,30^, logistic regression with elastic regularization (Elastic regression)^31^, Support Vector Machine (SVM)^32^, K-Nearest Neighbor (KNN) and regularized Random Forest^33,34^. To offer a comprehensive assessment of model performance we included a simple logistic regression model and a decision rule model aligning with current practices of assessing hypertension risk as references. Specifically, we included a logistic regression model using only age and BP as features, and a simple decision rule named “High normal BP rule”. The high normal BP rule predicts individuals with high normal BP (130/85 mmHg ≤ BP < 140/90 mmHg) at the baseline assessment as having 100% risk of incident hypertension at follow-up, and 0% otherwise. No neural networks algorithms were considered as they have been suggested to perform less favorable on tabular data^35^. For simplicity, features were included in the models without defining any interactions.

Hyperparameters were needed for several of the modelling methods. For XGBoost, we sampled 256 hyperparameter-combinations as candidates for cross-validation. We sampled 128 combinations each for both the Random Forest and the SVM modelling method. A grid search was used for Elastic regression and KNN models as these required less computational power. The hyperparameters, their ranges, search strategies and selected values are described in Supplementary Table S2.

Several steps were taken to minimize the risk of overfitting the data. First, we divided the available dataset randomly into a training and test set by a 7:3 ratio. Second, a fourfold cross-validation scheme was applied on the training set to select hyperparameters for our modelling methods. The combination of hyperparameters that produced the best mean out-of-fold performance during cross-validation was selected for each method separately. Using the selected hyperparameters, a final model for each method was fitted on the training set.

### Internal model validation

The final models were applied on the test set to evaluate performance. To account for variations in the test set, we applied bootstrapping with 1000 repetitions measuring the performance of all models on each bootstrapped test set. We summarized the performance measures by their means and 95% confidence intervals.

### Validation of external models

An important consideration to make seeing the already high number of developed hypertension risk models in the field is the need for another model^7^. Externally developed models may be used directly or easily adapted in some cases, making effective use of existing knowledge and reducing the probability of creating “research waste”. We address this concern by externally validating all applicable models we could find in the literature on our data. Perhaps equally important for hypertension risk models is that this contributes to assessing the generalizability of models, something which has been lacking for most hypertension risk models^4–7^.

We searched the literature for existing risk models that could be validated in our cohort by the following criteria: Using similar features to those available in the HUNT Study data; reporting model performance; and suitable for the 11-year follow-up period between baseline and outcome. From this, we found seven applicable risk models: Two clinical risk models for Chinese populations developed by Chien et al.^36^, The Framingham risk model developed using an American cohort by Parikh et al.^9^ and four more that were refitted versions of the Framingham risk model using either Korean or Iranian populations^37–39^. We validated these models upon the HUNT Study data, applying bootstrapping with 1000 repetitions to account for variations in the dataset. The external models and adaptations made on features are shown in Supplementary Note and Table S3.

An alternative to creating new risk models is to use a pre-existing model from literature. The use of the Framingham risk model was considered as an alternative. We choose the Framingham risk model as most applicable external models were adaptations of it, and it also had high resemblance with the risk models developed by Chien et al.^36^. We evaluated both the original version of the Framingham risk model and a recalibrated version tailored to the HUNT Study data. To perform recalibration, we followed Method 1 as described by Moons et al.^40^. As we did not have data meaningful for fitting time-to-event models, we used logistic regression to perform the recalibration. The performance of the original and recalibrated Framingham risk model was reported for comparison with the other developed models in this study^40,41^. Details on the original and recalibrated Framingham risk models are also presented in Supplementary Note and Table S3. We compare cohort summary data from the Framingham risk model development study and the HUNT Study in Supplementary Discussion and Supplementary Table S4.

### Performance indicators

To provide a complete view of model performance, we calculated multiple performance measures capturing discrimination, calibration, and clinical usefulness, as well as several measures commonly reported for ML methods. A particular emphasis was made on performance measures that did not transform predictions into binary outcomes, i.e., not requiring a probability threshold. We note that there is no such natural threshold value for hypertension risk modelling.

We evaluated discrimination performance by the A*rea Under the receiver operator Curve* (AUC), which is frequently used in risk modelling for hypertension and other binary outcomes^6,7,8^. We also estimated the models general performance by the scaled Brier score, which is a proper scoring function^8,42,43^. The scaled Brier score was applied as the common criteria for choosing the optimal hyperparameters during cross-validation.

Calibration was assessed graphically using smoothed calibration curves, and numerically summarized using the *Integrated Calibration Index* (ICI)^44,45^. The ICI measures the deviation of the smoothed calibration curve of a model versus a perfect, straight, diagonal calibration line, weighted by the distribution of the model’s predictions. A low ICI score suggests that the model is well-calibrated for the risk percentiles it frequently predicts in the dataset^44,45^.

Decision curve analysis was performed graphically by presenting the *Net Benefit* plot derived from the test set. The net benefit plot complements the calibration curve in assessing clinical usefulness of a risk model^8,46^. The benchmarks compared against in the Net Benefit plot was 1) predicting all as above the threshold of hypertension at follow-up, 2) predicting all as below the threshold of hypertension at follow-up and 3) the high normal BP decision rule previously described, where individuals with high normal BP were predicted as having 100% risk of being above threshold of hypertension at follow-up.

Lastly, we include auxiliary performance measures of discrimination that are frequently reported for ML models: The F1 measure, sensitivity, specificity, positive predictive value, negative predictive value, and the Matthews correlation coefficient^47^. For these, individual predictions need to be either below or above the hypertension threshold and not probabilities between zero and one. Thus, we assigned all predictions below the incidence rate of the training set (24.2%) as below the threshold, and all others as above.

### Feature importance

Assessing the feature importance of each feature for model performance can inform which features were necessary or unnecessary to include for obtaining a well-performing model. The motivation can be the derivation of an effective risk model using a subset of the original features. The subset of features may also have specific traits, such as being more cost-effective to obtain, accessible, or accurate in collection.

To estimate feature importance, we fitted Least Absolute Shrinkage and Selection Operator (LASSO) logistic regression models on the training set with increasing regularization penalty. As the penalty increased, the features coefficient sizes were tracked and we evaluated the LASSO model performance on the test set^48^. Features were included as linear effects without interactions.

To compare the feature importance found using the LASSO model, we calculated permutation importance for each feature with the XGBoost, K-Nearest Neighbor, SVM and Random Forest models on the test set using an adapted version of the procedure described by Fisher et al.^49^. In short, permutation importance for a feature was calculated as the change in performance for a model after randomly permuting that feature while keeping the remaining features fixed. The feature importance was reported as the mean permutation importance after repeating the procedure 1000 times. This provided some insight into how much each model relied on each feature for its performance on the test set.

An issue with permutation importance is bias introduced by correlated features^49^. To accommodate this, we calculated permutation importance on clusters of correlated features. We used hierarchical clustering of features with 1 – abs(X) as a distance metric, where X was the correlation between features. Using the max-distance criteria, clusters were merged until the distance between all pairs of clusters was 0.8 or higher.

### Subgroup analyses

To investigate the impact of using a threshold on continuous data such as systolic/diastolic BP to determine hypertension status, we performed two subgroup analyses with respect to baseline BP. Specifically, one subgroup was defined as individuals in the test set with BP below high-normal BP levels (< 130/85 mmHg) at baseline, and one subgroup as individuals with optimal BP (< 120/80 mmHg) at baseline. We compared summary statistics and report model performance calculated on these subgroups. In doing so, we also investigated the models’ ability to identify individuals with optimal BP levels at baseline that experienced a substantial increase in their BP to follow-up, 11 years later.

### Imputation of missing feature data

We performed a complete-case analysis, removing 5 087 records with missing feature entries from the dataset available from the HUNT Study. Ideally, multiple imputations (MI) should be used to handle feature data missing not completely at random^50^. The choice of doing a complete-case analysis instead of MI was the increased computational time in the main analysis. To assess the possible impact of performing MI, we applied the multiple imputation by chained equations (MICE)^51^ on the features in the original dataset containing all 22 939 individuals. Risk models were developed like in the main analysis but using only a subset of modelling methods with reduced hyperparameter searches. In total, we obtained four evaluations per modelling method: Models fitted on the training set with and without imputation, which were then evaluated on the test set with and without imputed records. See the Supplementary Method for more details.

### Selection bias due to loss to follow-up

In this study, 12 687 eligible participants in HUNT2 were lost to follow-up at HUNT3. Of these, 7 879 declined to participate, 2054 died, 2636 moved out of North Trøndelag County, and 118 had emigrated. A subset of 8 050 had complete feature data, meaning the effective loss to follow-up rate for our main analyses was 31%. To assess the degree of selection bias induced by loss to follow-up, we compared feature distributions between the lost and the included records and performed a post hoc sensitivity analysis. This analysis was performed by refitting elastic regression risk models similarly to the main analysis, but with each record weighted by their inverse probability of being among those lost to follow-up, as described by Howe et al.^52^. The probabilities were calculated using a pooled logistic regression model with all baseline features as linear features. Records from all 25 902 individuals who had complete data at baseline were used, with loss to follow-up as the outcome.

### Software

All data processing and analyses were performed in R (v. 4.1.2) using the RStudio IDE^53,54^. The *Tidyverse* (v. 1.3.1) package was used to process data and create figures, in combination with *ggExtra* (v. 0.10.1)*, cowplot* (v. 1.1.1) and *ggpattern* (v. 1.0.1)^55–58^. Summary statistics were calculated using *Skimr* (v. 2.1.5)^59^. Modelling was implemented using the *caret* (v. 6.0–94)*, glmnet* (v. 4.1–8), *mice* (v. 3.16.0) and *doSNOW* (v. 1.0.20) packages^60–62^. Performance measures were calculated using *caret* (v. 6.0–94) and *dcurves* (v. 0.4.0) packages^60,63^. The smoothed calibration curve was computed using the *loess* function with default parameters, and hierarchical clustering by the *hclust* function, both from the *stats* (v 4.1.2) base package in R^53^.

## Results

Summary statistics for the cohort are provided in Supplementary Table S5. There were significant differences in all features when stratified by outcome status. The 11-year hypertension incidence rate was 24.41%.

**Figure 1:**
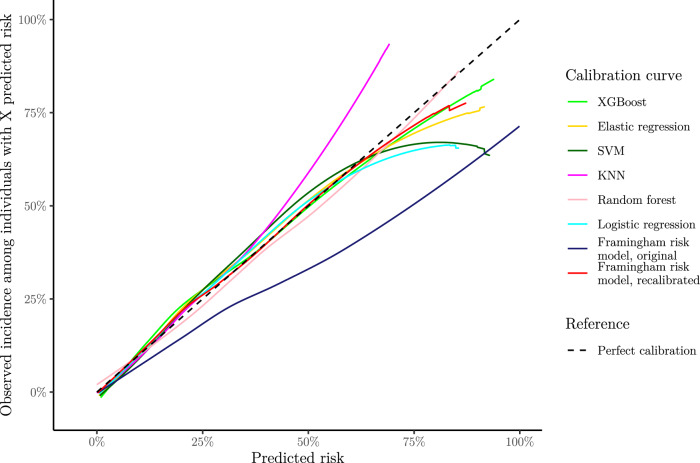
Smoothed calibration curves for the test set. Calibration curves close to the dashed reference line exhibit an elevated level of agreement between its predictions and the observed incidence in the test set. Curves are shown as pointwise mean curves calculated by bootstrapping. *KNN* K-nearest neighbors, *SVM* support vector machines, *XGBoost* eXtreme gradient boosting.

**Figure 2:**
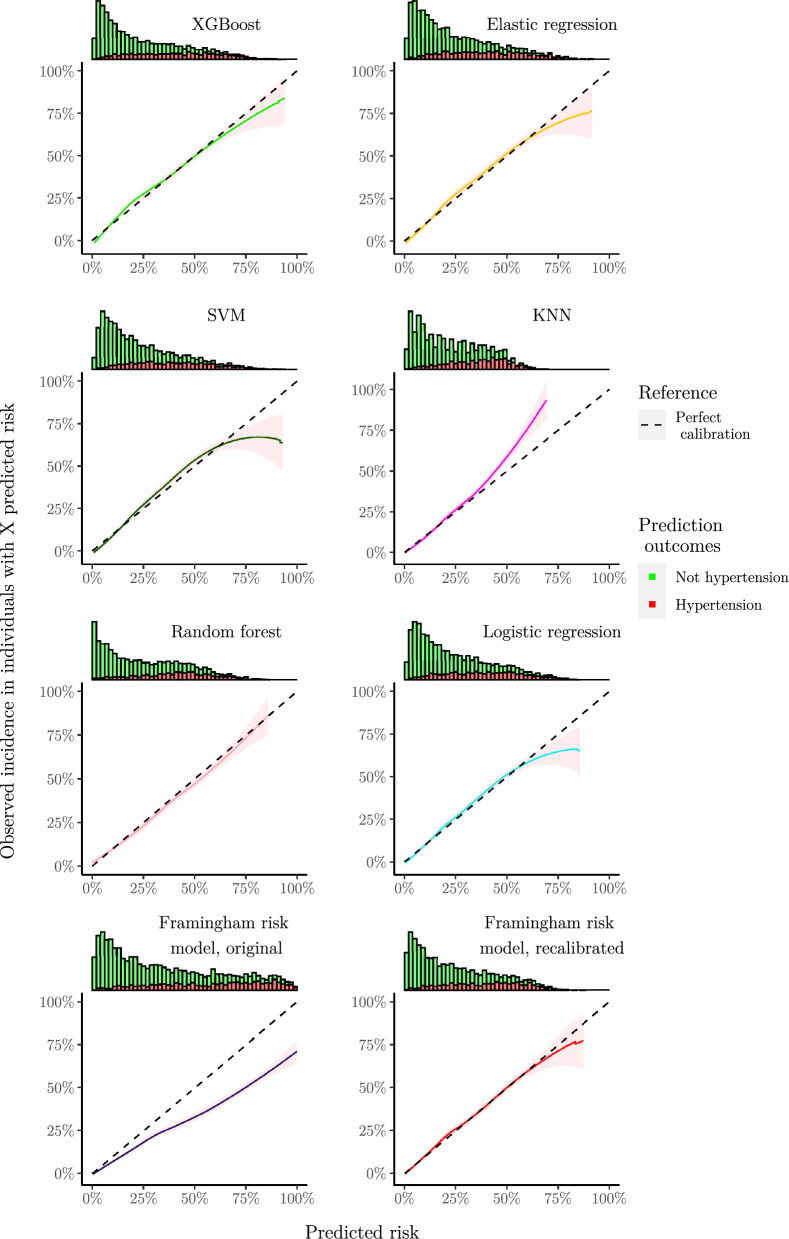
Calibration curves with histogram of predictions above. The histogram is colored by proportion of incidence. Curves are shown as pointwise mean curves with red shaded 95% confidence interval calculated by bootstrapping. *KNN* K-nearest neighbors, *SVM* support vector machines, *XGBoost* eXtreme gradient boosting.

**Figure 3:**
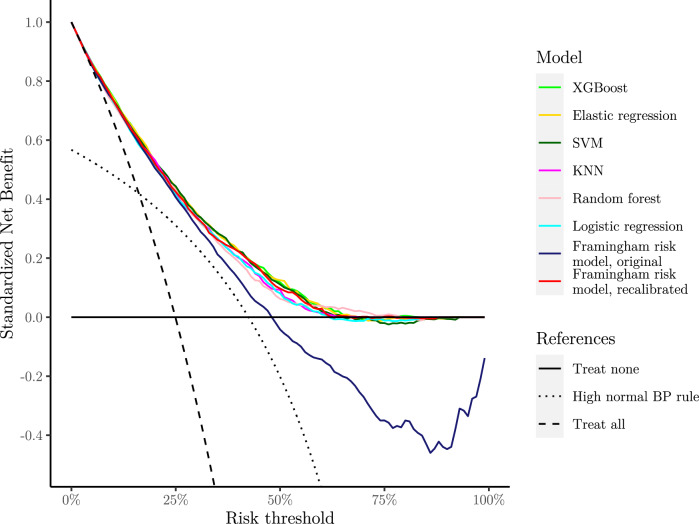
Decision curves of all models. Net benefit was standardized to have a max value of 1. Curves are shown as pointwise mean curves calculated by bootstrapping. *BP* blood pressure, *KNN* K-nearest neighbors, *SVM* support vector machines, *XGBoost* eXtreme gradient boosting.

**Figure 4:**
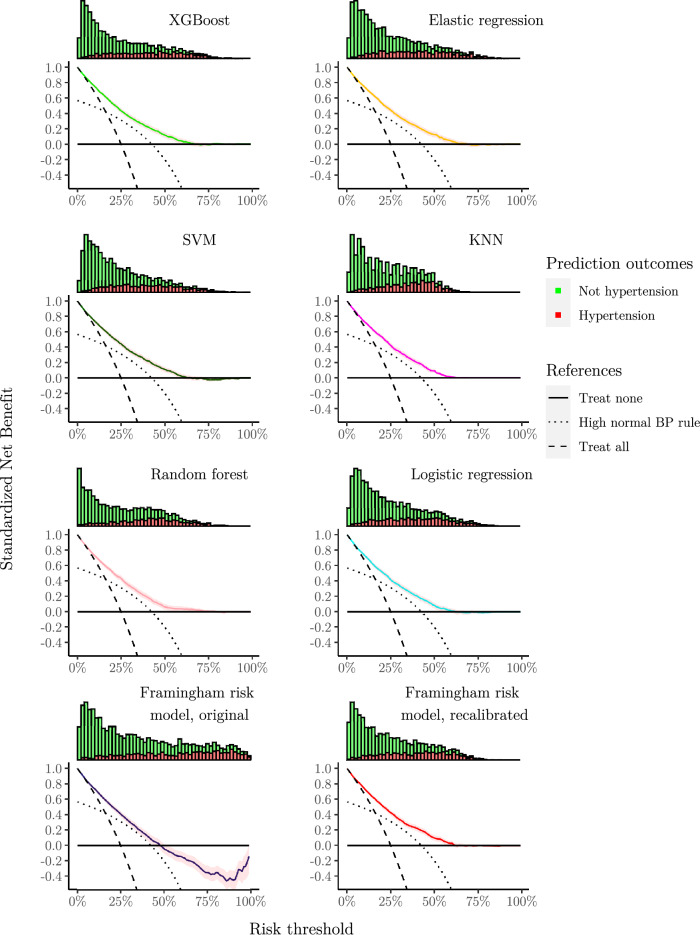
Decision curves with histogram of predictions above. The histogram is colored by the proportion of incidence. Net Benefit is standardized to have a max value of 1. Curves are shown as pointwise mean curves with red shaded 95% confidence interval calculated by bootstrapping. *KNN* K-nearest neighbors, *SVM* support vector machines, *XGBoost* eXtreme gradient boosting.

Applying the fitted models on the test set, we obtained the results given in Table 1. Discrimination was good for all models, except the High normal BP rule. XGBoost, Elastic regression and SVM performed slightly better than the others. Most developed models were well-calibrated, as shown by low ICI and their calibration curves in Figs. 1 and 2. The KNN model underestimated risk at predictions higher than 35%, whereas the remaining models slightly overestimated risk at predictions higher than 60%. The developed models had similarly shaped decision curves, shown in Figs. 3 and 4, in which models scoring higher on discrimination displayed a slightly higher net benefit across all thresholds. All ML models improved upon the decision curve references. Auxiliary performance measures are presented in Supplementary Table S6.

**Table 1:** Model results achieved on test set, n = 5 356.

Models	AUC (↑)	Scaled brier (↑)	ICI (↓)
ML			
XGBoost	**0.795 [0.782, 0.808]**	**0.204 [0.181, 0.225]**	0.016 [0.009, 0.025]
Elastic regression	**0.795 [0.781, 0.807]**	**0.204 [0.182, 0.223]**	0.016 [0.009, 0.025]
SVM	0.792 [0.779, 0.804]	0.198 [0.177, 0.217]	0.021 [0.012, 0.030]
KNN	0.786 [0.772, 0.799]	0.186 [0.169, 0.202]	0.024 [0.015, 0.034]
Random forest	0.778 [0.763, 0.791]	0.181 [0.157, 0.202]	0.017 [0.009, 0.027]
References			
Logistic regression	0.780 [0.766, 0.792]	0.181 [0.160, 0.201]	0.014 [0.007, 0.022]
High normal BP rule*	0.656 [0.641, 0.670]	–	–
External			
Framingham risk model, original	0.786 [0.773, 0.799]	0.078 [0.037, 0.114]	0.115 [0.104, 0.125]
Framingham risk model, recalibrated	0.786 [0.773, 0.799]	0.192 [0.170, 0.211]	**0.010 [0.005, 0.017]**

Best observed mean performances are in [bold].

Performance obtained applying the fitted models on the test set. Reported as mean and 95% confidence interval after bootstrapping. The symbols (↑) and (↓) indicates increasing or decreasing values as improved performance, respectively.

*Scaled Brier score and ICI is omitted for ‘High normal BP rule’ as calibration is not meaningful when predictions are either 0% or 100% risk.

*AUC* area under the receiver–operator curve, *ICI* integrated calibration index, *KNN* K-nearest neighbors, *ML* machine learning, *SVM* support vector machines, *XGBoost* eXtreme gradient boosting.

### Validation of external models

The Framingham risk model performed comparably with newly developed models on discrimination, but overestimated risk as shown by the higher ICI and calibration curve, see Table 1, and Figs. 1 and 2. The effect of poor calibration can be seen in its decision curve in Figs. 3 and 4 where net benefit was consistently lower, and even negative for higher predictions. After a simple recalibration of the Framingham risk model that preserved discrimination performance, the ICI score and calibration curve show a well-calibrated model that had a decision curve close to the best of the new models based on results from the test set, see Table 1.

Results for all six external models validated on the whole dataset are provided in Table 2. In summary, all models displayed good discrimination, yielding bootstrapped AUC means of 0.787–0.789 for the Framingham risk model and its refitted versions, and 0.768 for the Chinese risk model. Of all external models, the Framingham risk model was the only model displaying a positive scaled Brier score. All external models had high ICI scores, i.e., poor calibration on the HUNT Study cohort. The calibration curves of the external models shown in Supplementary Fig. S2 indicate that all external models exaggerated the predicted risk.

**Table 2:** External model results achieved on the complete dataset, n = 17 852.

External models	AUC (↑)	Scaled Brier (↑) ^a^	ICI (↓)
Framingham risk model, original^9^	0.788 [0.781, 0.795]	**0.080 [0.059, 0.103]**	**0.115 [0.109, 0.121]**
Chinese clinical risk model^36^	0.768 [0.760, 0.775]	− 1.344 [− 1.413, − 1.281]	0.515 [0.509, 0.522]
Chinese clinical risk model, from individuals without diabetes at baseline^36^	0.761 [0.752, 0.769]	− 1.474 [− 1.546, − 1.409]	0.537 [0.530, 0.543]
KoGES model^37^	**0.789 [0.782, 0.796]**	− 0.113 [− 0.143, − 0.081]	0.204 [0.198, 0.210]
TLGS model^38^	0.787 [0.779, 0.794]	− 0.265 [− 0.300, − 0.229]	0.248 [0.242, 0.255]
CAVAS model^39^	0.788 [0.782, 0.795]	− 0.049 [− 0.073, − 0.023]	0.201 [0.195, 0.207]
F-CAVAS model^39^	0.788 [0.781, 0.795]	− 0.056 [− 0.080, − 0.030]	0.204 [0.198, 0.210]

Best observed mean performances are in [bold].

Performance obtained applying the fitted models on the whole dataset. Reported as mean and 95% confidence interval after bootstrapping. The symbols (↑) and (↓) indicates increasing or decreasing values as improved performance, respectively.

^a^A negative Scaled Brier score implies predicted probabilities were on average worse than using the outcome rate, i.e., 24.41%, as a prediction. This is likely due to the poor calibration exhibited by all models.

*AUC* area under the receiver–operator curve, ICI integrated calibration index.

### Feature importance

**Figure 5:**
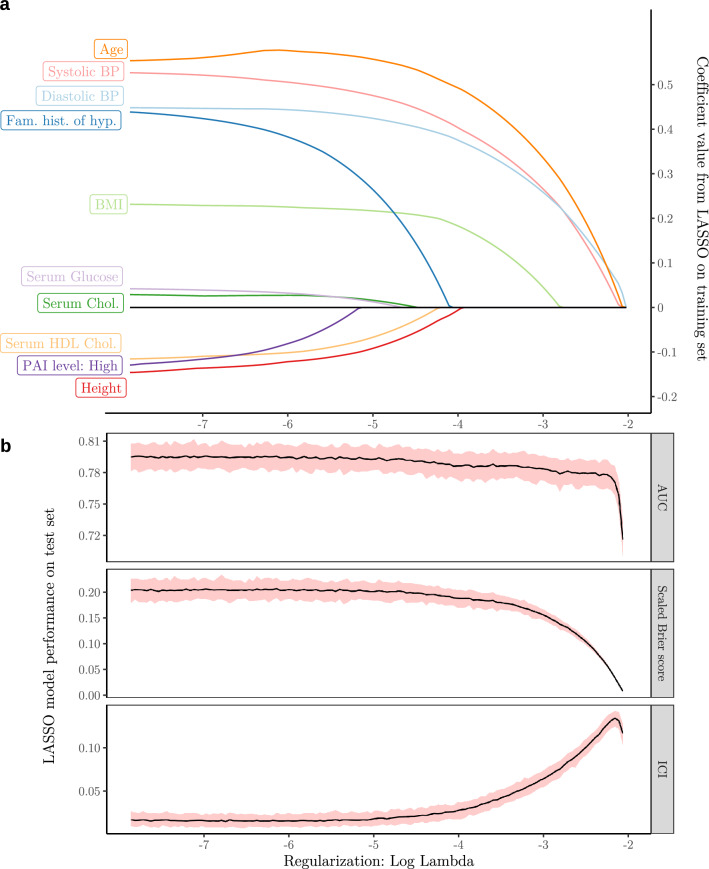
(**a**) Coefficient sizes in least absolute shrinkage and selection operator (LASSO) regression fitted on the training set with increasing regularization. Only the 10 last features to be zeroed out are shown. (**b**) The performance of the LASSO regression model on the test set as regularization was increased. Curves are shown as pointwise mean curves with red shaded 95% confidence interval calculated by bootstrapping. *AUC* area under the receiver-operator curve, *BMI* body mass index, *BP* blood pressure, *Chol* cholesterol, *Fam. hist. of hyp.* family history of hypertension, *HDL* high-density lipid, *ICI* integrated calibration index. *Log* natural logarithm, *PAI* physical activity indicator.

In LASSO regression, the coefficients for ‘age’, ‘systolic BP’ and ‘diastolic BP’ required far higher penalties to be zeroed out compared to other features. In addition, while noting that all numerical features were standardized, their coefficients were larger compared to all others, meaning that they also had the highest effect on predictions. Other notable features were ‘body mass index (BMI)’, ‘height’, ‘family history of hypertension’, and blood serum markers such as ‘triglycerides’, ‘cholesterol’ and ‘high-density lipoprotein cholesterol’. Notably, as the LASSO penalty was increased, discrimination performance was stable while the calibration of the model deteriorated more quickly. Coefficients and performance of the LASSO model on the test set under increasing regularization penalty are shown in Fig. 5.

**Figure 6:**
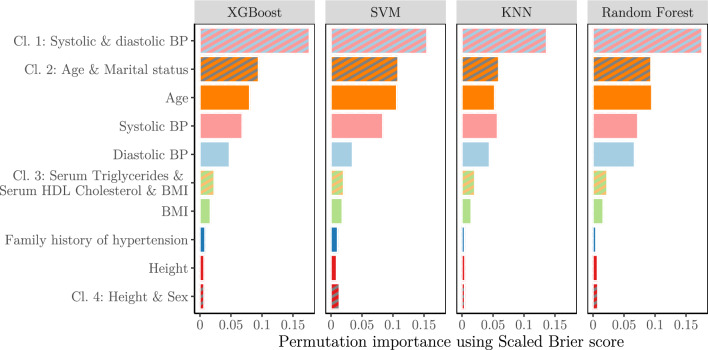
Permutation importance calculated for XGBoost, SVM, KNN and random forest models. The importance of a feature or cluster was determined as the average decrease in Scaled Brier score on the test set when the feature or cluster was permuted. Features are colored following Fig. 5—Panel A, with gray for ‘Sex’ and ‘Marital status’, and combined colors for clusters. Irrelevant features or clusters, defined as those with a mean decrease of less than 0.004 in Scaled Brier score, were left out for conciseness. Features in clusters were permuted simultaneously. *BMI* body mass index, *BP* blood pressure, *Cl*. # feature cluster #, *HDL* high-density lipid, *KNN* K-nearest neighbors, *SVM* support vector machines, *XGBoost* eXtreme gradient boosting.

The permutation importance largely agreed with the LASSO importances. A minority of features, namely ‘age’, ‘systolic BP’ and ‘diastolic BP’, were highly emphasized in all models. In addition, ‘BMI’, ‘family history of hypertension’ and ‘height’ were notable, but far lower compared to the former three. In hierarchical clustering of features by correlation, we identified five clusters of features: (1) ‘Systolic BP’ and ‘diastolic BP’, (2) ‘age’ and ‘marital status’, (3) ‘triglycerides’, ‘HDL cholesterol’, and ‘BMI’, (4) ‘height’ and ‘sex’, and (5) ‘creatinine’ and ‘estimated glomerular filtration rate’. The importance was higher for each cluster than the summed importances of their individual member features. Clusters 1–2 had far greater importance than clusters 3–5, again emphasizing the importance of ‘age’, ‘systolic BP’ and ‘diastolic BP’. Permutation importance calculated for the ML models using scaled Brier score is shown in Fig. 6, and using AUC shown in Supplementary Fig. S3.

### Subgroup analyses

Summary statistics for the individuals in the test set with BP below high-normal BP (< 130/85 mmHg) are provided and compared versus individuals in the test set with high-normal BP (130/85 mmHg ≤ BP < 140/90 mmHg), at baseline, in Supplementary Table S7. Notably, there were significant differences between the two groups in all included features except ‘physical activity’ and ‘marital status’. The incidence rate was only 16% among those with BP below high-normal BP at baseline versus 42% among those with high-normal BP at baseline.

Most models displayed good discrimination and calibration on the subgroup , see Table 3. The ML models outperformed the reference models, with XGBoost being the best. However, the improvement was minor compared to the linear model produced with Elastic regression. Of note, the Framingham risk model had good discrimination, although recalibration was still needed for its predictions to be considered well-calibrated. In general, discrimination performance was lower in the subgroup analyses compared to the main analyses using the complete cohort, but calibration was similar.

**Table 3:** Model results achieved on records within the test set that had normal BP or lower (< 130/85 mmHg) at baseline, n = 3 573.

Models	AUC (↑)	Scaled Brier (↑)	ICI (↓)
ML			
XGBoost	**0.778 [0.758, 0.796]**	**0.135 [0.108, 0.160]**	0.017 [0.010, 0.025]
Elastic regression	0.774 [0.753, 0.792]	0.132 [0.106, 0.158]	0.017 [0.009, 0.026]
SVM	0.768 [0.747, 0.787]	0.125 [0.099, 0.151]	0.015 [0.009, 0.024]
KNN	0.761 [0.741, 0.779]	0.121 [0.096, 0.143]	**0.013 [0.006, 0.020]**
Random forest	0.753 [0.732, 0.773]	0.105 [0.075, 0.133]	0.024 [0.014, 0.034]
Reference			
Logistic regression	0.751 [0.729, 0.771]	0.111 [0.086, 0.134]	**0.013 [0.007, 0.022]**
External			
Framingham risk model, original	0.762 [0.741, 0.782]	0.061 [0.017, 0.101]	0.066 [0.055, 0.077]
Framingham risk model, recalibrated	0.762 [0.741, 0.782]	0.122 [0.098, 0.144]	0.014 [0.006, 0.023]

Best observed mean performances are in [bold].

Performance obtained applying the fitted models on the test set after excluding individuals with high normal BP (≥ 130/85 mmHg) at baseline. Reported as mean and 95% confidence interval after bootstrapping. The symbols (↑) and (↓) signify increasing or decreasing values as improved performance, respectively. Note that the ‘High normal BP’ rule was not included as the subgroup does not contain any individuals with high normal BP at baseline.

*AUC* area under the receiver–operator curve, ICI integrated calibration index, *KNN* K-nearest neighbors, *ML* machine learning, SVM support vector machines, *XGBoost* eXtreme gradient boosting.

Summary statistics for individuals in the test set with optimal BP (< 120/80 mmHg), are shown and compared versus individuals in the test set with normal BP or higher (120/80 mmHg ≤ BP < 140/90 mmHg), at baseline in Supplementary Table S8. There were significant differences between the two groups in all included features except estimated glomerular filtration rate, family history of hypertension, physical activity, and marital status. The incidence rate was only 10% among those with optimal BP at baseline versus 32% among those with normal BP or higher at baseline.

Most models displayed good discrimination and calibration on the subgroup, see Table 4. Based on performance, we obtained the same ranking of models as for the main analysis. However, the net improvement for the XGBoost models compared to remaining models was more pronounced. In short, the best performing models were effective in identifying individuals with optimal BP at baseline that developed hypertension in the 11 years until follow-up.

**Table 4:** Model results achieved on records within the test set that had optimal BP (< 120/80 mmHg) at baseline, n = 1 809.

Models	AUC (↑)	Scaled Brier (↑)	ICI (↓)
ML			
XGBoost	**0.783 [0.747, 0.817]**	**0.091 [0.055, 0.124]**	0.020 [0.010, 0.032]
Elastic regression	0.768 [0.730, 0.804]	0.084 [0.053, 0.113]	0.021 [0.012, 0.032]
SVM	0.757 [0.721, 0.794]	0.071 [0.038, 0.104]	0.021 [0.012, 0.031]
KNN	0.753 [0.716, 0.79]	0.072 [0.039, 0.105]	**0.016 [0.009, 0.025]**
Random forest	0.750 [0.712, 0.787]	0.061 [0.011, 0.107]	0.025 [0.013, 0.037]
Reference			
Logistic regression	0.728 [0.688, 0.766]	0.051 [0.025, 0.076]	0.022 [0.013, 0.033]
External			
Framingham risk model, original	0.755 [0.714, 0.792]	0.066 [0.023, 0.103]	0.029 [0.019, 0.040]
Framingham risk model, recalibrated	0.755 [0.714, 0.792]	0.071 [0.047, 0.093]	0.025 [0.014, 0.037]

Best observed mean performances are in [bold].

Performance obtained applying the fitted models on the test set after excluding individuals with high normal BP (≥ 130/85 mmHg) at baseline. Reported as mean and 95% confidence interval after bootstrapping. The symbols (↑) and (↓) signify increasing or decreasing values as improved performance, respectively. Note that the ‘High normal BP’ rule was not included as the subgroup does not contain any individuals with high normal BP at baseline.

*AUC* area under the receiver–operator curve, *ICI* integrated calibration index, *KNN* K-nearest neighbors, *ML* machine learning, *SVM* support vector machines, *XGBoost* eXtreme gradient boosting.

### Imputation of missing feature data

Feature distributions remained similar when individuals with missing feature data were included in the dataset, see Supplementary Table S9. Although there were significant changes for the distribution age, sex and education, the differences between data sets were small. Using the MICE procedure for MI, model performance increased on the imputed test set (n = 6 883) compared to the complete test set (n = 5 356), as shown in Supplementary Table S10. However, this seems to be an artifact more related to the data than the models. Model performance was similar when compared on the same data, regardless of whether the model was fitted using complete training set (n = 12 496) or training set after imputation with MICE (n = 16 056). In short, the removal of individuals with missing feature data in the main analysis seems unlikely to have affected the relative ranking of the models.

### Selection bias due to loss to follow-up

When comparing feature distributions between records lost to follow-up and those included in the main analyses, all 18 features had statistically significant differences, except for 'BMI' and 'non-fasting serum glucose', as shown in Supplementary Table S11. This suggests that the group lost to follow-up are different from the group who remained in the study.

In a sensitivity analysis, we repeated the development of the elastic regression model as in the main analysis, using data weighted by the inverse probability of loss to follow-up. We used elastic regression for this post hoc analysis as it has a low computational burden and produced one of the top performing models in the main analysis. In the weighted sensitivity analysis, overall mean performance on the test set improved by < 0.05% for AUC and < 0.20% for scaled Brier score, while worsening by < 1.00% for ICI compared to the results from the main analysis. In conclusion, although there are significant differences between those who were lost to follow-up and those included in our study, the sensitivity analysis suggests negligible effects on model performance due to selection bias due to loss to follow-up.

## Discussion

In this study, we used a large cohort to create several risk models for incident hypertension, the first study using data from Norway. The developed models displayed good discrimination on the test set, indicating that individuals at risk of developing hypertension within 11 years could be well identified. Based on calibration curves and ICI, the predictions provided by most of the models were well-calibrated, and all models displayed higher net benefit for their decision curves than the decision curve benchmarks. Subgroup analyses show that the models were effective in identifying individuals that had optimal or normal BP at baseline but experienced a substantial increase in BP and developed hypertension before the 11-year follow-up.

We found ML models to perform well and improve upon the reference regression model and decision rule used. Although the performance measures worsened slightly in subgroup analyses, the overall pattern was similar with ML models excelling over references. On another remark, while the XGBoost and SVM methods are capable of learning non-linear effects of its input features, the Elastic regression model using only linear effects performed similarly. While this is not proof of absence for non-linear effects in our dataset, it suggests that linear models are sufficient to obtain a practically optimal risk prediction model for this cohort from the HUNT Study.

The external risk models validated in our dataset had good discrimination, comparable to the developed models. However, as expected, all external models had poor calibration on the HUNT Study data. Indeed, external risk models often require recalibration to new cohorts and do not perform as well as internal models^40^. After recalibration of the Framingham risk model, the model performed comparably with some of the newly developed ML models, providing an AUC only one percentage point lower than the best-performing ML model whilst having excellent calibration. Considering that only a single parameter was estimated for the recalibration, the use of an external model seems like an attractive option compared to developing new risk models.

The 95% prediction interval for the AUC of a new hypertension risk model has been shown to be quite wide in a recent meta-analysis, i.e., 0.660–0.865 for traditional models and 0.547 – 0.943 for ML models, and influenced by both study design and cohort characteristics^7^. Thus, knowing how well new models perform requires context provided within the study itself. By using reference models, we can obtain an indication if a superficially well-performing model achieves its performance from aspects related to the model or the data. This is exemplified by the High normal BP rule we included, which, although simple in its construction, produces AUC scores on par with several risk models presented in the field, including several ML models^7^. The simple reference model using only age and BP, as well as the external model validations, serves as context as more complex models are developed, e.g., using ML. In addition, we see that comparisons must be made on the same data, as using different datasets may affect performance measures. This is exemplified by the difference in performance for the Framingham risk model on the complete dataset versus the test set, and the differences seen in our sensitivity analysis using MI on missing feature data. Hence, our results emphasize the importance of including reference models for comparison, and comparing on the same data.

In our analyses, we employ decision curves to evaluate the net benefit of the developed, reference, and external models in clinical practice. Yet, to aptly interpret a decision curve, we would be required to define a range of reasonable threshold probabilities for determining when interventions are warranted^64^. However, there are no established threshold probabilities for hypertension, and Vickers et al. emphasize that one should not use the decision curve to select one either^64^. Similar works developing risk models for incident hypertension have used different thresholds in calculating performance measures or characterize the risk level of individuals, e.g., 4%, 5%, 8%, 10%, 15%, 16%, 20%, etc.^9,36,39,65–70^. The incidence rate could be an option; however, the incidence rate has been seen to vary considerably in studies developing risk models. While we did not define a reasonable range of threshold probabilities, the XGBoost, Elastic regression and SVM models show the highest and similar benefit across most thresholds, indicating that they should be preferred over the other alternatives^64^.

In the feature importance analyses, ‘age’ and ‘systolic/diastolic BP’ at baseline were particularly important. This was also shown by the well-performing reference model, which had an AUC only two percentage points lower than the best ML model on the test set. Other emphasized features were ‘BMI’, ‘height’, ‘family history of hypertension’, and various blood serum markers, but to a far lower degree than ‘age’ and ‘systolic/diastolic BP’. Both LASSO and permutation importance showed similar results. This is in line with other works in the field^11,14,15^. Further, the notion that few features were required for obtaining well-performing models has been demonstrated in previous work, albeit mostly with traditional models^36,71–73^.

With the use of more complex data sources, such as Electronic Health Records (EHRs) or genetic profiles, knowledge-driven feature selection can be replaced or supplemented by data-driven feature selection, which ML could prove to be more flexible and capable for than traditional models in risk modelling. Examples include risk models developed by Datta et al. and Kanegae et al. for EHRs^13,14^, and Niu et al. for genetics^12^. Further, important features for model performance could be used to identify new risk factors relevant for understanding hypertension development.

ML for hypertension risk prediction has received increased interest in later years, with multiple risk models being developed^6,7^. In this study, we applied ML simply as a modelling alternative to traditional methods for prognostic risk prediction models. Although no improvement was found for non-linear models compared to linear models in our main analysis, non-linear ML models excelled somewhat in identifying individuals with a large increase in BP (> 20/10 mmHg). Early identification and initiation of preventative treatment for these individuals could be valuable, as such large increases in BP is associated with double risk of ischemic heart disease and stroke, regardless of sex and age. In our view, ML techniques in hypertension diagnostics should focus on identifying individuals that would be likely to benefit from earlier intervention, e.g. lifestyle modifications, to prevent or at least delay the onset of hypertension.

Although ML may have potential for hypertension risk modelling, there are several drawbacks that increase the required workload and computational cost. First, to overcome the “black box” nature of many ML methods, one must rely on auxiliary methods. Second, the no-free-lunch theorem^74^ implies that multiple ML methods should be considered, as we do not know in advance which method that works best. Third, with the increased capacity of many ML methods for learning data patterns, ensuring proper internal validation during model development is important^50^, which has been lacking for hypertension risk models^7^. Fourth, adequate attribution of any performance improvement requires comparison to alternatives, e.g., less complex models, to eliminate alternative explanations. Finally, the application of ML does not by itself guarantee improved performance, as demonstrated in our study’s main analysis. While many of these items are relevant for risk modelling in general, they become particularly important to ensure transparency and scientific value when applying ML on health data. Further analyses of ML risk models for hypertension by external researchers are often not possible as the models are not made available in the literature^7^. In addition, reproduction of the models on the same data is unlikely due to general data protection regulations that prevent open access to data from health surveys or medical records for external researchers.

## Limitations

One study limitation arises from the long time between baseline to follow-up combined with the modifiability of features. Although we included 18 features representing well-established risk factors of hypertension, many of these are highly modifiable and our models do not account for lifestyle changes during follow-up. For instance, we know that body mass and prevalence of diabetes increased in the study population between 1995 and 2008, while total cholesterol and the prevalence of daily smoking decreased^22,23^. In addition, the risk profile in HUNT2, which the developed risk models are based on, was collected about 3 decades ago. Although the risk factors remain the same, the composite risk profiles may have changed since then.

Another limitation of our work is that we were restricted to a moderate set of features. Firstly, in other works where ML has been applied, ML risk models for incident hypertension has been found to produce high AUC scores in studies with large number (> 100) of features, such as those using electronic health records^13,75^. In studies where a moderate number of features were included, the results were more mixed: ML exceled over logistic regression or Cox regression in some studies^12,14,17^ but performed similarly based on AUC in others^10,11,15,71^. The impact on calibration and clinical usefulness is unclear, as both have been given limited attention in studies developing ML models. Secondly, we did not employ any feature-selection prior to inclusion into our models. The motivation was that we only had a moderate number of features, that several of the methods already had built-in capabilities for regularization, and that it would add more complexity to the analyses as we would have to decide on one of many feature selection strategies and methods. Thirdly, we did not address the accessibility of features that were included in our analyses. This would be relevant in the case a model is considered for adoption into clinical practice, and can be included as an additional consideration in decision analysis^64^. Finally, important risk factors related to hypertension progression such as diet and alcohol use were absent from the features^1^. Although highly modifiable, the inclusion of features related to these could allow more accurate risk prediction for the developed models. In the literature, Nusinovici et al. and Kanegae et al. found alcohol to be of little importance relative to systolic BP^11,14^, and Kadomatsu et al. found that alcohol did not improve the mean AUC in a model with age, smoking and BP^73^. Neither assessed features related to diet. Chowdhury et al. found both vegetable/fruit consumption and alcohol ranked as among the least-important features for their models using multiple ranking methods^15^. Nevertheless, features related to diet and alcohol are often considered for hypertension risk prediction^7^.

Lastly, we did not evaluate our models on an external dataset, which should be done before clinical adoption^8,40^. In this study, we focused on developing risk models and assess the possible advantages of using ML. Acknowledging the large variation in AUC between studies in the field^7^, it is likely that study conditions such as study design, features, and cohort characteristics could affect model performance. Further, the generalizability of the models may also be limited as our cohort consisted of an ethnically homogenous population from a country with high standards of living. Also, we note that there were fewer men than women in the complete cohort, with incidence rates differing. While men represented 38% of the participants in the complete cohort, they represented 43% of the outcomes, i.e., men were more at risk of developing hypertension compared to women.

## Conclusion

We developed models for predicting the 11-year risk of incident hypertension in a Norwegian cohort considering multiple alternative methods including both ML and traditional risk models. The models were mostly well-calibrated and successful in identifying individuals at risk of developing hypertension, even among individuals that had optimal or normal BP at baseline. The risk models developed using XGBoost and Elastic regression performed similarly and slightly better than the others in our analyses. However, the improvements upon the reference model using only age and BP or the recalibrated external Framingham risk model were small. In feature importance analysis, age, systolic and diastolic BP was emphasized as particularly important for risk prediction, followed by BMI, height, and family history of hypertension. We found that linear effects were sufficient for obtaining a well-performing model compared to non-linear modelling for our data. Further, few features were needed for a well-performing model, shown by the reference model and feature importance. The externally developed Framingham risk model performed well on our cohort after recalibration. Our work demonstrates the importance of including reasonable reference models when evaluating risk models, as well as the benefit of considering existing models from the literature.

### Supplementary Information

Supplementary Information.

## Data Availability

The data that support the findings of this study are available from HUNT Research Centre but are currently not publicly available. The data can be obtained upon approval from Regional Committee on Medical and Health Research Ethics of Norway and HUNT Research Centre. For more information see: www.ntnu.edu/hunt/data. To request the data from this study, please contact the corresponding author. Availability of developed models. To allow for scientific dissemination by external researchers, we share the developed models and resources for using them at https://github.com/filsch/hypertension_prediction_models_hunt_study.
