# Supplementary material for: Development of risk models of incident hypertension using machine learning on the HUNT study data
Source: Sci Rep. 2024 Mar 7;14:5609. doi: 10.1038/s41598-024-56170-7 (PMC10920790; doi:10.1038/s41598-024-56170-7)
Supplement: Supplementary file 1 — Supplementary Information. [file 41598_2024_56170_MOESM1_ESM.pdf]

Supplementary Item S1

TRIPOD Checklist: Prediction Model Development

Section/Topic	Item	Checklist Item	Page
**Title and abstract**			
Title	1	Identify the study as developing and/or validating a multivariable prediction model, the target population, and the outcome to be predicted.	1, 2
Abstract	2	Provide a summary of objectives, study design, setting, participants, sample size, predictors, outcome, statistical analysis, results, and conclusions.	1, 2, 3
**Introduction**			
Background and objectives	3a	Explain the medical context (including whether diagnostic or prognostic) and rationale for developing or validating the multivariable prediction model, including references to existing models.	2, 3
	3b	Specify the objectives, including whether the study describes the development or validation of the model or both.	1, 2
**Methods**			
Source of data	4a	Describe the study design or source of data (e.g., randomized trial, cohort, or registry data), separately for the development and validation data sets, if applicable.	3, 4
	4b	Specify the key study dates, including start of accrual; end of accrual; and, if applicable, end of follow-up.	3, 4
Participants	5a	Specify key elements of the study setting (e.g., primary care, secondary care, general population) including number and location of centres.	3, 4
	5b	Describe eligibility criteria for participants.	3
	5c	Give details of treatments received, if relevant.	-
Outcome	6a	Clearly define the outcome that is predicted by the prediction model, including how and when assessed.	3, 4
	6b	Report any actions to blind assessment of the outcome to be predicted.	-
Predictors	7a	Clearly define all predictors used in developing or validating the multivariable prediction model, including how and when they were measured.	4, Table S1
	7b	Report any actions to blind assessment of predictors for the outcome and other predictors.	-
Sample size	8	Explain how the study size was arrived at.	3, 4, Figure S1
Missing data	9	Describe how missing data were handled (e.g., complete-case analysis, single imputation, multiple imputation) with details of any imputation method.	3, 4, 7
Statistical analysis methods	10a	Describe how predictors were handled in the analyses.	4, 5
	10b	Specify type of model, all model-building procedures (including any predictor selection), and method for internal validation.	4, 5
	10d	Specify all measures used to assess model performance and, if relevant, to compare multiple models.	6, 7
Risk groups	11	Provide details on how risk groups were created, if done.	-
**Results**			
Participants	13a	Describe the flow of participants through the study, including the number of participants with and without the outcome and, if applicable, a summary of the follow-up time. A diagram may be helpful.	3, 4, Figure S1, Table S5
	13b	Describe the characteristics of the participants (basic demographics, clinical features, available predictors), including the number of participants with missing data for predictors and outcome.	3, 4, S7, Tables S9, S11
Model development	14a	Specify the number of participants and outcome events in each analysis.	Figure S2, Tables S7-9, S11
	14b	If done, report the unadjusted association between each candidate predictor and outcome.	-
Model specification	15a	Present the full prediction model to allow predictions for individuals (i.e., all regression coefficients, and model intercept or baseline survival at a given time point).	20 (online), Table S5
	15b	Explain how to the use the prediction model.	20 (online), Table S3
Model performance	16	Report performance measures (with CIs) for the prediction model.	8, 9, 10
**Discussion**			
Limitations	18	Discuss any limitations of the study (such as nonrepresentative sample, few events per predictor, missing data).	13, 14
Interpretation	19b	Give an overall interpretation of the results, considering objectives, limitations, and results from similar studies, and other relevant evidence.	11-14
Implications	20	Discuss the potential clinical use of the model and implications for future research.	12-14
**Other information**			
Supplementary information	21	Provide information about the availability of supplementary resources, such as study protocol, Web calculator, and data sets.	20
Funding	22	Give the source of funding and the role of the funders for the present study.	20

We recommend using the TRIPOD Checklist in conjunction with the TRIPOD Explanation and Elaboration document.

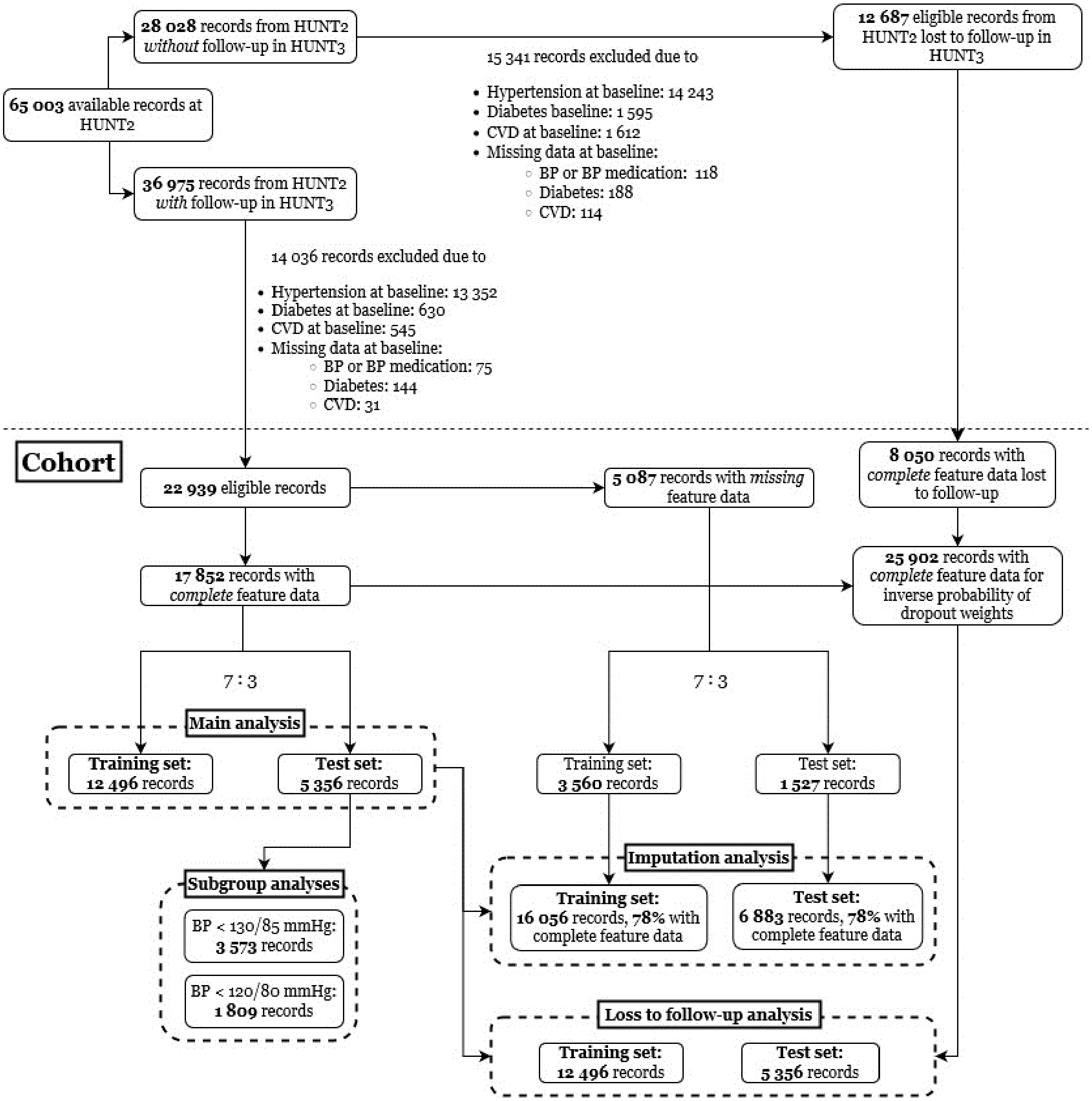

Supplementary Figure S1. Flow of data used in analyses. Individuals in the HUNT Study data supplied one record each. The “7 : 3” refers to splits of the data done by random sampling in a 7:3 ratio. The selection criteria ‘BP < …/…’ refer to baseline blood pressure for individuals in the test set. Note, some records fulfilled multiple exclusion criteria. In addition, some individuals have withdrawn their consent to participate in the HUNT Study, hence the available number of records being fewer than those reported in earlier cohort profiles.
‘BP’: Blood pressure, ‘CVD’: Cardiovascular disease.

Supplementary Table S1. The variable names that were used to construct features, as named in HUNT databank.

**Variable names (units)**	**Constructed using the following variable names in HUNT Databank**
Age (years)	PartAg@NT2BLQ1
Height (cm)	Hei@NT2BLM
BMI [a] (m/kg^2)	Bmi@NT2BLM
Systolic blood pressure (mmHg)	BPSystMn23@NT2BLM
Diastolic blood pressure (mmHg)	BPDiasMn23@NT2BLM
Serum Creatinine (µmol/L)	SeCreaCorr@NT2BLM
Serum Cholesterol (mmol/L)	SeChol@NT2BLM
Serum high-density lipid Cholesterol (mmol/L)	SeHDLChol@NT2BLM
Serum Triglyceride (mmol/L)	SeTrig@NT2BLM
Non-fasting Serum Glucose (mmol/L)	SeGluNonFast@NT2BLM
Sex	.
Estimated Glomerular Filtration Rate Stage	GFREstMDRD@NT2BLM
Family history of hypertension	BPHigFamNon@NT2BLQ2, PHigMothEv@NT2BLQ2, BPHigFathEv@NT2BLQ2, BPHigBrotEv@NT2BLQ2, BPHigSistEv@NT2BLQ2, BPHigChiEv@NT2BLQ2
Family history of CVD	CarInfFam1@NT2BLQ1
Smoking status	SmoStat@NT2BLQ1
Socio-economic status: Highest level of education attained.	Educ@NT2BLQ1
Physical activity, measured in Physical Activity Intelligence (PAI).	ExeLigDuLY@NT2BLQ1, ExeHarDuLY@NT2BLQ1, Sex
Exclusion criteria; Diabetes and CVD at baseline	DiaEv@NT2BLQ1, SeGluNonFast@NT2BLM, CarInfEv@NT2BLQ1
Hypertension status at baseline	BPDiasMn23@NT2BLM, BPSystMn23@NT2BLM, BPMedCu@NT2BLQ1
Hypertension status at follow up	BPSystMn23@NT3BLM, BPDiasMn23@NT3BLM, BPMedSiEffEv@NT3CvdQ, BPMedEv@NT3BLQ1

Info on variables in the HUNT databank can be found at https://hunt-db.medisin.ntnu.no/hunt-db/#/.

[a] Decorrelated with respect to height in the HUNT databank.

'BMI': Body Mass Index, 'CVD': cardiovascular disease.

Supplementary Table S2. Hyperparameters selected for different methods in cross-validation.

**Modelling method, method name** [a]	**Hyperparameter** [a]	**Value selected**	**Grid**	**Search strategy**
XGBoost, 'xgbtree'	'mrounds'	915	{1, 2, ..., 1000}	Uniform random sampling/draw, n=256 trials
	'max depth'	2	{1, 2, ..., 10}	
	'eta'	0.02023	[0.001, 0.6]	
	'gamma'	1.54503	[0, 10]	
	'colsample_bytree'	0.32728	[0.3, 0.7]	
	'min_child_weight'	3	{0, 1, ..., 20}	
	'subsample'	0.80038	[0.25, 1]	
Elastic regression, 'elastic'	'alpha'	0.51613	{0, 0.1, ..., 1}	All combinations
	'lambda'	0.00458	{exp(-1), exp(-2), ..., exp(-9)}	
SVM w/ polynomial kernel 'svmp'	'degree'	1	{1, 2, 3}	Uniform random sampling/draw, n=128 trials
	'scale'	0.01201	[10^(-5), 10^(2)]	
	'C'	643.962	[2^(-5), 2^(10)]	
K-Nearest Neighbor, 'knn'	'K'	130	{1, 2, 3, 4, 5, 6, 7, 8, 9, 10, 12, 15, 20, 30, 40, 50, 60, 70, 80, 90, 100, 110, 120, 130, 140, 150, 160, 170, 180, 190, 200, 210, 220}	All values
Regularized Random Forest, 'RRF'	'mtry'	14	{1, 2, ..., 22}	Uniform random sampling/draw, n=128 trials
	'coefReg'	0.77257	[0, 1]	
	'coefImp'	0.02289	[0, 1]	

Grids were derived from default options for random search used in the 'caret' package. Hyperparameter and model naming follow the conventions of the 'caret' package. Grid dimensions reported as sets of numbers, e.g., {a, b, c, d} or intervals, e.g., [x, y]. Numbers were rounded to five decimals.

[a] Name given in the caret software package.

'exp': the exponential function, 'KNN': K-Nearest Neighbors, 'SVM': Support Vector Machines, 'XGBoost': eXtreme Gradient Boosting.

Supplementary Note

To create risk predictions using the external Framingham risk model and its refitted versions, two or three adaptations had to be made to the models due to mismatch in feature-definitions. Instead of the number of parents with hypertension used in the Framingham, CAVAS, and F-CAVAS risk models, we used family history of hypertension. We accommodated this by multiplying the model

coefficient with the average number of parents with hypertension, if any, for the three models. This was 1.6394 for the Framingham risk model, and 1.108 for the CAVAS and F-CAVAS models. The feature parental hypertension in TLGS and KoGES models was simply replaced with family history of hypertension. Further, 'Formerly daily smoker' and 'Never' in the HUNT Study was encoded as 'No', and 'Daily smoker' encoded as 'Yes' for feature 'Current smoker' for all models. Lastly, to accommodate the risk horizon of 11 years in this study, we calculated the risk of an event within 11 years, i.e., using t=11 in the Weibull equations. All external model equations used are shown in Supplementary Table S3.

For recalibration of the Framingham risk model to the HUNT Study data, we used the linear predictor of the adapted Framingham risk model as a single predictor in a logistic regression model. We fixed the slope to 1 and only estimated an intercept, i.e., an offset, from the HUNT Study data, following Method 1 described by Moons et al.[1]. See Supplementary Table S3 for the exact risk equation used and adaptations made.

Supplementary Table S3. External risk models with adaptations and the recalibration of the Framingham risk model to the HUNT Study data.

**Model**	**Model used for 11-year risk,** y_{11}**:**
Framingham risk model, from Parikh et al[2]. *	$y_{11} = 1 - \exp(-\exp(z))$, $z = \frac{\ln(11) - x}{0.8769}$, $x = 22.9495 - 0.15641 \times \text{'Age'} - 0.20293 \times \text{'Female'}$ $-0.05933 \times \text{'Systolic BP'} - 0.12847 \times \text{'Diastolic BP.'}$ $-0.19073 \times \text{'Smoking'} - 0.03388 \times \text{'BMI'}$ $-0.16612 \times 1.6394 \times \text{'Fam. hist. of hyp.''}$ $+0.00162 \times \text{'Age'} \times \text{'Diastolic BP'}$
Framingham risk model, recalibrated to the HUNT Study data.	$y_{11} = \frac{1}{1 + \exp(-s)}$, $s = \alpha + z$, $\alpha = -0.3367$, z as above, α estimated from the training set.
Chinese clinical risk model, from Chien et al.[3].	$y_{11} = 1 - \exp(-\exp(z))$, $z = \frac{\ln(11) - x}{0.592}$, $x = 8.173 - 0.011 \times \text{'Age'} + 0.124 \times \text{'Female'}$ $-0.029 \times \text{'Systolic BP'} - 0.014 \times \text{'Diastolic BP.'}$ $-0.043 \times \text{'BMI'}$
Chinese clinical risk model, from individuals without diabetes at baseline, from Chien et al.[3].	$y_{11} = 1 - \exp(-\exp(z))$, $z = \frac{\ln(11) - x}{0.59}$, $x = 8.141 - 0.009 \times \text{'Age'} + 0.154 \times \text{'Female'}$ $-0.029 \times \text{'Systolic BP'} - 0.014 \times \text{'Diastolic BP.'}$ $-0.048 \times \text{'BMI'}$

KoGES model, from Lim et al.[4].	$y_{11} = 1 - \exp(-\exp(z)),\quad z = \frac{\ln(11) - x}{1.0805},$ $x = 30.3547 - 0.2838 \times \text{'Age'} - 0.2084 \times \text{'Female'} - 0.0642 \times \text{'Systolic BP'} - 0.2033 \times \text{'Diastolic BP.'} - 0.2912 \times \text{'Smoking'} - 0.0665 \times \text{'BMI'} - 0.0875 \times \text{'Fam. hist. of hyp.'} + 0.0031 \times \text{'Age'} \times \text{'Diastolic BP'}$
TLGS model, from Koohi et al.[5].	$y_{11} = 1 - \exp(-\exp(z)),\quad z = \frac{\ln(11) - x}{0.6401},$ $x = 17.0917 - 0.1279 \times \text{'Age'} + 0.1355 \times \text{'Female'} - 0.0322 \times \text{'Systolic BP'} - 0.1113 \times \text{'Diastolic BP.'} - 0.1912 \times \text{'Smoking'} - 0.0294 \times \text{'BMI'} - 0.2302 \times \text{'Fam. hist. of hyp.'} + 0.0013 \times \text{'Age'} \times \text{'Diastolic BP'}$
CAVAS model, from Namgung et al.[6]. *	$y_{11} = 1 - \exp(-\exp(z)),\quad z = \frac{\ln(11) - x}{0.6908},$ $x = 13.544 - 0.0801 \times \text{'Age'} - 0.0874 \times \text{'Female'} - 0.0366 \times \text{'Systolic BP'} - 0.0605 \times \text{'Diastolic BP.'} - 0.0277 \times \text{'BMI'} - 0.1906 \times 1.108 \times \text{'Fam. hist. of hyp.'} + 0.0008 \times \text{'Age'} \times \text{'Diastolic BP'}$
F-CAVAS model, from Namgung et al.[6]. *	$y_{11} = 1 - \exp(-\exp(z)),\quad z = \frac{\ln(11) - x}{0.6908},$ $x = 13.5468 - 0.0803 \times \text{'Age'} - 0.0827 \times \text{'Female'} - 0.0366 \times \text{'Systolic BP'} - 0.0606 \times \text{'Diastolic BP.'} + 0.0146 \times \text{'Smoking'} - 0.0277 \times \text{'BMI'} - 0.1902 \times 1.108 \times \text{'Fam. hist. of hyp.'} + 0.0008 \times \text{'Age'} \times \text{'Diastolic BP'}$

To accommodate the risk horizon of 11 years, t=11 is used in the Weibull regression for all models. For the feature ‘Smoking’, the levels ‘Formerly daily smoker’ and ‘Never’ in the HUNT Study were both encoded as ‘No’, and ‘Daily smoker’ encoded as ‘Yes’. For features ‘Female’, ‘Smoking’ and ‘Fam. hist. of hyp.’, levels ‘Yes’ and ‘No’ were set as 1 or 0, respectively, when used in model equations. Remaining features were set as their numeric value.

* The feature ‘Number of parents with hypertension’ in the models was replaced with ‘Family history of hypertension’ and its coefficient multiplied by the average number of parents with hypertension in the respective development cohorts, i.e., 1.6394 in the Framingham model, and 1.108 in the KoGES models.

‘BMI’: Body Mass Index, ‘BP’: Blood pressure, ‘Fam. hist. of hyp.’: Family history of hypertension.

Supplementary Discussion

The overestimation of risk made by the Framingham risk model might be due to differences in the study and cohorts baseline characteristics, shown in Supplementary Table S4. The notable aspects are that the incidence rate, systolic blood pressure and smoking were highly different between the

two cohorts. However, in the Framingham Offspring Study, an individual could supply multiple records in the data set due to multiple follow ups, until they either presented with hypertension at an assessment or being censured at the end of study. This means that while 1717 individuals were included in the study, a total of 5814 records were used. With 796 outcomes, only 13.7% of the included records had hypertension as the outcome in contrast to an incidence rate per individual of 45%. For systolic blood pressure and smoking, the inclusion of multiple follow ups means that the baseline data characteristics might not be representative of the 5814 records that were used, e.g., individuals may have stopped smoking or experienced changes to their systolic BP between assessments.

Parental, or familial, history of hypertension was different between the studies, which may have to do with differences in recording of that variable. In the Framingham Offspring Study, the parent's history of hypertension was recorded as part of another study, meaning the information is more precise than that recorded in the HUNT Study, which relied on participants reporting their parents' hypertension history via questionnaires. However, parental/familial history of hypertension was associated with increased risk in the Framingham risk model so, in isolation, we would expect lower risk estimates for the HUNT Study data as it had a lower rate. This was not the case, as the Framingham risk model overestimated risk for the HUNT Study data. Lastly, we note that there was a higher proportion of women included in the HUNT Study. In short, based on the study and cohort characteristics, it is not clear why the Framingham risk model overestimated risk for the HUNT Study cohort.

Supplementary Table S4. Study and data characteristics for the Framingham Offspring Study cohort used to develop the Framingham risk model and the HUNT Study cohort used in this study.

**Study characteristics**	**Framingham Offspring Study**	**HUNT Study II**
Cohort origin	New England, USA	Trøndelag, Norway
Study collection year	1979 – 1998	1995 – 1997
Follow-up time	Median 4 years	Median 11 years
Number of follow-ups	Multiple follow-ups, up to 5 times	Single follow-up
Study size	1 717 individuals, 5814 records	17 852 individuals/records
Incidence rate	Individuals: 45 %. Records: 14%	24 %
Hypertension definition	Systolic BP ≥ 140 mmHg, diastolic BP ≥ 90 mmHg, or use of blood pressure–lowering medications.	Systolic BP ≥ 140 mmHg, diastolic BP ≥ 90 mmHg, or use of blood pressure–lowering medications.
**Baseline data characteristics:**		
Age	42.2 (9.6)	42.53 (11.68)
Women	54.1 %	62 %

Systolic blood pressure	116 (11.1)	122.85 (9.96)
Diastolic blood pressure	75 (7.5)	73.83 (7.76)
Current smoker	Yes: 35.2 %	Daily: 28 %
Parental/familial history of hypertension	Any parent: 94.3 %	Any first-degree relative: 36 %
Body Mass Index	25.1 (4.1)	25.38 (3.63)

Reported as mean (standard deviation) for numerical data, and percentage for categorical data. 'BMI': Body Mass Index, 'BP': Blood pressure, 'CVD': Cardiovascular disease.

Supplementary Method

The original dataset made available from the HUNT Study had 5 087 (22%) records with missing entries in their feature data. However, missing entries were low for most features, see Supplementary Table S9. Only 'family history of hypertension' exceeded 10%, with 'family history of CVD', 'socio-economic status' and 'physical activity' being the only others exceeding 1%. In summary, 4 441 individuals missed one entry, 568 missed two entries, 68 missed three, nine missed four, and one missed five. In testing for differences between the datasets with and without those that had missing feature entries, we found significant differences on means or proportions in 'age', 'sex', and 'socioeconomic status'. However, among these, all differences were small, and only 'socioeconomic status' had any missing values with 1.4% missing entries.

Ideally, the missing feature entries would have been imputed using multiple imputation[7]. Instead, we removed them from the dataset for our main analysis and used only individuals that had complete data available. This was motivated by reducing the time and computational burden imposed by the multiple imputation procedure. As a sensitivity analysis, we used Multiple Imputation by Chained Equations (MICE) to impute our data and develop risk models. To compare changes in performance, we calculated performance measures four ways: Models fitted with and without imputed data were evaluated on the test set with and without individuals with imputed entries.

We reduced the hyperparameter search space and chose a subset of methods that we empirically found to be faster to fit. Using the MICE procedure on the original dataset with missing feature entries, we performed model development using the subset of methods and hyperparameters. We sampled individuals with missing data by a 7:3 ratio, adding them to the already defined training and testing set used in the main analysis. Model development was done by selecting hyperparameters with four-fold cross-validation. We applied MICE within the cross-validation routine to avoid data leakage, i.e., using only the training folds to learn the imputation parameters at each iteration. We generated 20 imputed versions of datasets and used 10 imputation iterations whenever MICE was applied. After model development, MICE was applied again to learn parameters from the full training set to impute the test set. Results from the four-way evaluations are shown in Supplementary Table S10, where the results from the main analysis are also included.

The pattern was reminiscent to results from the main analysis: Most models perform well, with ML models outperforming the logistic regression model and the high normal BP rule. The Framingham risk model was again worse than the ML models but better than the reference models on discrimination. However, recalibration was required to achieve acceptable calibration for the Framingham risk model.

Supplementary Table S5. Feature distributions of the complete data set used in the main analysis, stratified by outcome status.

**Feature**	**Levels**	**All, n=17 852**	**Not hypertension at year 11, n=13 494 (76%)**	**Hypertension at year 11, n= 4 358 (24%)**	t / χ^2, P value [a]
Age (years)	-	42.53 (11.68)	40.53 (11.23)	48.71 (10.86)	P < 0.0001*
Height (cm)	-	170.59 (8.74)	170.74 (8.69)	170.12 (8.87)	P < 0.0001*
BMI (m/kg^2)	-	25.38 (3.63)	24.98 (3.42)	26.62 (3.95)	P < 0.0001*
Systolic blood pressure (mmHg)	-	122.85 (9.96)	121.15 (9.89)	128.1 (8.21)	P < 0.0001*
Diastolic blood pressure (mmHg)	-	73.83 (7.76)	72.47 (7.55)	78.03 (6.84)	P < 0.0001*
Serum Creatinine (µmol/L)	-	66.67 (12.96)	66.06 (12.63)	68.54 (13.79)	P < 0.0001*
Serum Cholesterol (mmol/L)	-	5.56 (1.13)	5.45 (1.11)	5.92 (1.11)	P < 0.0001*
Serum high-density lipid Cholesterol (mmol/L)	-	1.42 (0.38)	1.44 (0.38)	1.38 (0.38)	P < 0.0001*
Serum Triglyceride (mmol/L)	-	1.49 (0.93)	1.42 (0.89)	1.72 (1.03)	P < 0.0001*
Non-fasting Serum Glucose (mmol/L)	-	5.1 (0.84)	5.05 (0.82)	5.26 (0.88)	P < 0.0001*

**Feature**	**Levels**	**All, n=17 852**	**Not hypertension at year 11, n=13 494 (76%)**	**Hypertension at year 11, n= 4 358 (24%)**	t / χ^2, P value [a]
Sex	Male	6 748 (38%)	4 884 (36%)	1 864 (43%)	P < 0.0001*
	Female	11 104 (62%)	8 610 (64%)	2 494 (57%)	
Estimated Glomerular Filtration Rate Stage	Rate < 90 ml/min.:	11 890 (67%)	9 352 (69%)	2 538 (58%)	P < 0.0001*
	Rate ≥ 90 ml/min.:	5 962 (33%)	4 142 (31%)	1 820 (42%)	
Family history of hypertension	Yes	6 432 (36%)	4 504 (33%)	1 928 (44%)	P < 0.0001*
Family history of CVD	Yes	6 730 (38%)	4 700 (35%)	2 030 (47%)	P < 0.0001*
Smoking Status (Daily)	Never	8 237 (46%)	6 425 (48%)	1 812 (42%)	P < 0.0001*
	Formerly	4 678 (26%)	3 343 (25%)	1 335 (31%)	
	Currently	4 937 (28%)	3726 (28%)	1 211 (28%)	
Socioeconomic Status: Highest level of education attained	Secondary school	3 843 (22%)	2 506 (19%)	1 337 (31%)	P < 0.0001*
	Upper secondary school	6 969 (39%)	5 253 (39%)	1 716 (39%)	
	High school	2 063 (12%)	1 752 (13%)	311 (7%)	
	Higher education, < 4 years	3 036 (17%)	2 435 (18%)	601 (14%)	
	Higher education, ≥ 4 years	1 941 (11%)	1 548 (11%)	393 (9%)	
Physical Activity, measured in Physical Activity Intelligence (PAI).	Low (< 50 PAI)	6 088 (34%)	4 370 (32%)	1 718 (39%)	P < 0.0001*
	Medium (50-99 PAI)	4 726 (26%)	3 514 (26%)	1 212 (28%)	
	High (≥ 100 PAI)	7 038 (39%)	5 610 (42%)	1 428 (33%)	
Marital status	Never married	4 453 (25%)	3 900 (29%)	553 (13%)	P < 0.0001*
	Married	12 127 (68%)	8 677 (64%)	3 450 (79%)	

Feature	Levels	All, n=17 852	Not hypertension at year 11, n=13 494 (76%)	Hypertension at year 11, n= 4 358 (24%)	t / χ^2, P value [a]
	Divorced	1 272 (7%)	917 (7%)	355 (8%)	

Reported as mean (standard deviation) for numerical features, and count (percentage) for categorical features. P-values were rounded to four decimals.

* Significant after applying Holm's step-down correction with α = 0.05.

[a] Two-tailed Welch t-test for a difference in mean value for numerical features or chi-square test for a difference in proportions in categorical features. Each individual feature was tested as the group of individuals free of hypertension versus the group with hypertension, at follow-up.

Supplementary Table S6. Auxiliary performance measures calculated on the test set.

**Models**	**F1 score**	**Sensitivity**	**Specificity**	**PPV**	**NPV**	**MCC**
XGBoost	0.556 [0.536, 0.576]	0.745 [0.719, 0.769]	0.690 [0.676, 0.706]	0.444 [0.423, 0.465]	0.891 [0.879, 0.902]	0.382 [0.355, 0.407]
Elastic regression	**0.561 [0.541, 0.581]**	0.747 [0.722, 0.770]	0.697 [0.682, 0.711]	0.449 [0.429, 0.470]	0.893 [0.881, 0.903]	**0.389 [0.362, 0.414]**
SVM	**0.561 [0.540, 0.580]**	0.739 [0.715, 0.763]	0.703 [0.689, 0.717]	**0.452 [0.431, 0.473]**	0.890 [0.880, 0.901]	**0.389 [0.363, 0.413]**
KNN	0.549 [0.528, 0.569]	0.755 [0.731, 0.780]	0.670 [0.656, 0.685]	0.431 [0.411, 0.452]	0.892 [0.881, 0.903]	0.371 [0.346, 0.395]
Random forest	0.549 [0.528, 0.568]	0.798 [0.774, 0.820]	0.632 [0.617, 0.647]	0.418 [0.398, 0.437]	0.904 [0.893, 0.915]	0.372 [0.347, 0.396]
Logistic regression	0.546 [0.525, 0.565]	0.738 [0.714, 0.761]	0.680 [0.666, 0.694]	0.433 [0.412, 0.454]	0.887 [0.876, 0.898]	0.366 [0.340, 0.389]
High normal BP rule	0.485 [0.463, 0.506]	0.567 [0.542, 0.592]	**0.744 [0.730, 0.757]**	0.423 [0.400, 0.446]	0.838 [0.827, 0.850]	0.285 [0.258, 0.312]

Framingham risk model	0.533 [0.515, 0.551]	**0.868 [0.850, 0.886]**	0.540 [0.525, 0.556]	0.385 [0.367, 0.402]	**0.925 [0.915, 0.935]**	0.356 [0.333, 0.377]
Framingham risk model recalibrated	0.549 [0.529, 0.569]	0.750 [0.727, 0.773]	0.674 [0.659, 0.689]	0.433 [0.411, 0.453]	0.891 [0.880, 0.901]	0.370 [0.344, 0.395]

Reported as mean and 95% confidence interval after bootstrapping. The incidence rate of the training set, 24.2%, was used as probability threshold to calculate the measures.
'KNN': K-Nearest Neighbors, 'MCC': Matthews Correlation Coefficient, 'NPV': Negative Prediction Value, 'PPV': Positive Prediction Value, 'SVM': Support Vector Machines, 'XGBoost': eXtreme Gradient Boosting.

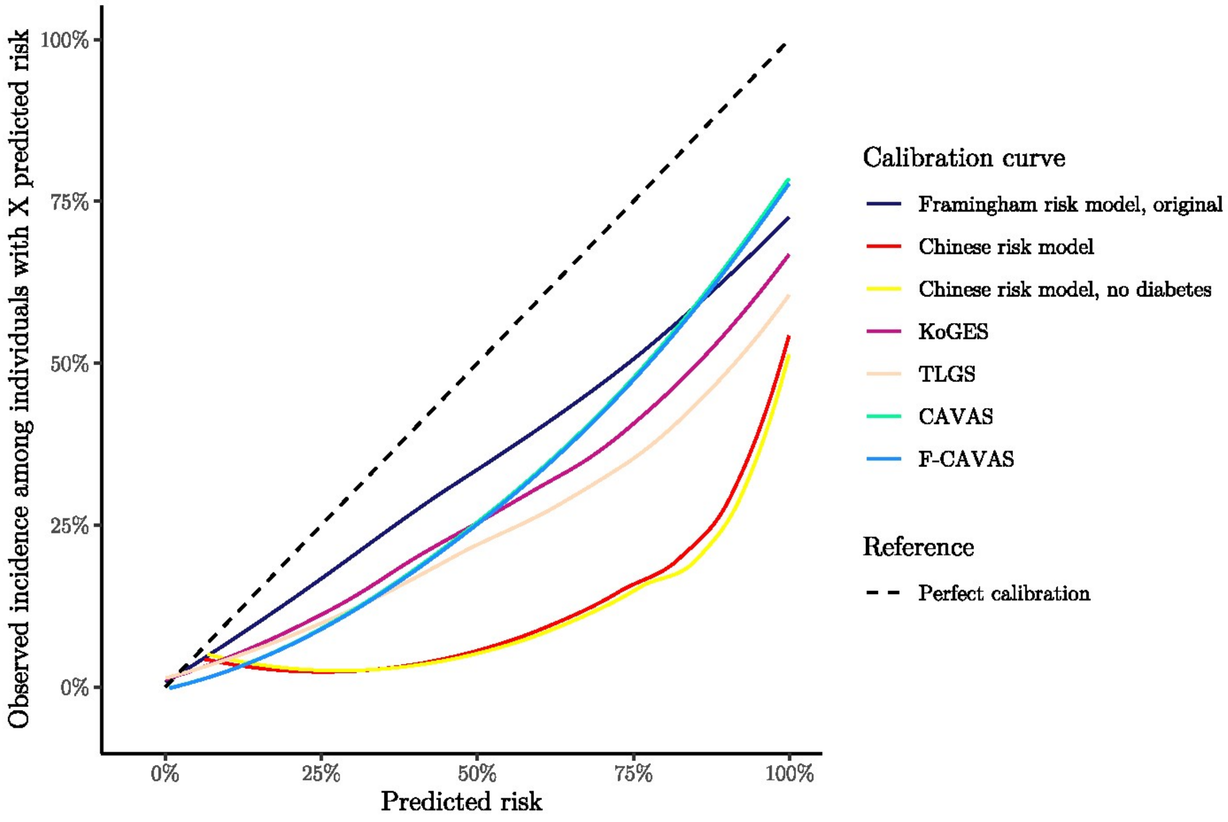

Supplementary Figure S2. Smoothed calibration curves calculated on the complete dataset. Calibration curves close to the dashed reference line exhibit an elevated level of agreement between its predictions and the observed incidence in the complete dataset. Curves are shown as pointwise mean curves calculated by bootstrapping.

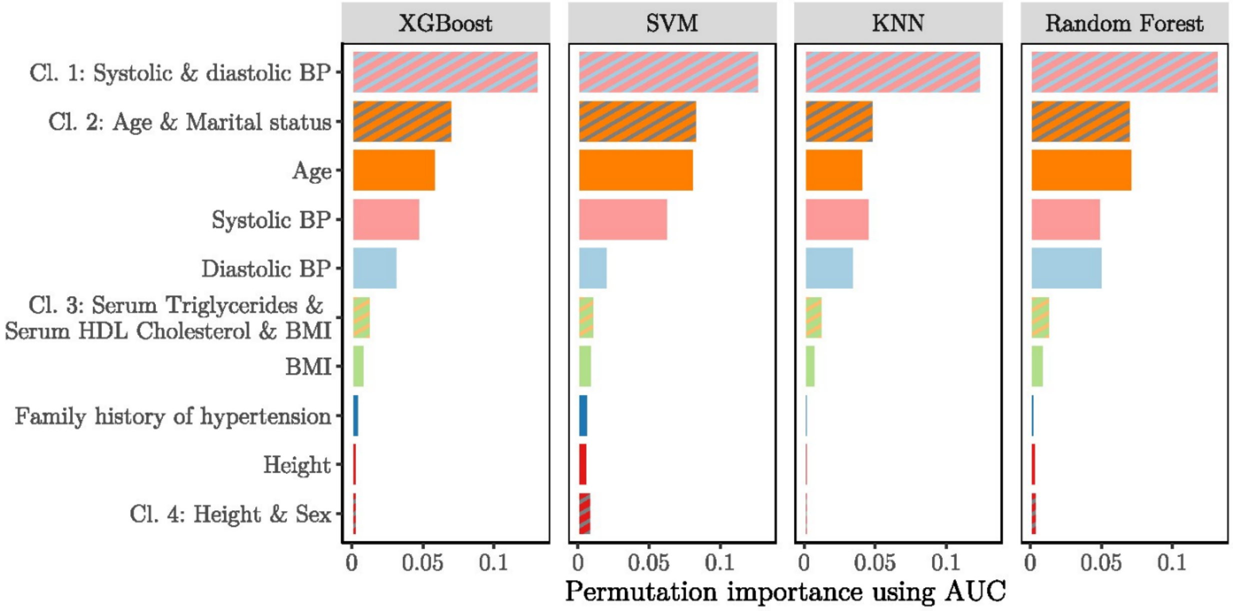

Supplementary Figure S3. Permutation importance calculated for XGBoost, SVM, KNN and Random Forest models. The importance of a feature or cluster was determined as the mean decrease in AUC on the test set when the feature or cluster was randomly permuted. Features in clusters were permuted simultaneously. Coloring follows Figure 5 – Panel A, with gray for 'Sex' and 'Marital status', and combined colors for clusters. Irrelevant features or clusters, defined as those with a mean decrease of less than 0.004 in Scaled Brier Score, were left out for conciseness. 'BMI': Body Mass Index, 'BP': Blood pressure, 'Cl. #': Feature cluster #, 'HDL' High-density lipids, 'KNN': K-Nearest Neighbors, 'SVM': Support Vector Machines, 'XGBoost': eXtreme Gradient Boosting.

Supplementary Table S7. Feature and outcome distributions of records in the test set stratified by individuals having normal BP or lower (<130/85 mmHg) versus high normal BP (130/85 mmHg ≤ BP < 140/90 mmHg) at baseline.

**Feature**	**Levels**	Individuals with normal BP or lower, n=3 573	Individuals with high normal BP, n=1 783	t / χ^2, P value [a]
Age (years)	-	41.31 (11.23)	44.92 (12.21)	P < 0.0001*
Height (cm)	-	169.69 (8.33)	172.66 (9.18)	P < 0.0001*
BMI (m/kg^2)	-	24.93 (3.56)	26.24 (3.6)	P < 0.0001*
Systolic blood pressure (mmHg)	-	117.78 (7.75)	133.35 (3.87)	P < 0.0001*

Feature	Levels	Individuals with normal BP or lower, n=3 573	Individuals with high normal BP, n=1 783	t / χ^2, P value [a]
Diastolic blood pressure (mmHg)	-	71.38 (6.86)	79.2 (6.68)	P < 0.0001*
Serum Creatinine (µmol/L)	-	65.06 (12.36)	69.99 (13.07)	P < 0.0001*
Serum Cholesterol (mmol/L)	-	5.46 (1.11)	5.84 (1.16)	P < 0.0001*
Serum high-density lipid Cholesterol (mmol/L)	-	1.45 (0.37)	1.37 (0.37)	P < 0.0001*
Serum Triglyceride (mmol/L)	-	1.39 (0.86)	1.7 (0.99)	P < 0.0001*
Non-fasting Serum Glucose (mmol/L)	-	5.02 (0.82)	5.25 (0.92)	P < 0.0001*
Sex	Male	1072 (30%)	967 (54%)	P < 0.0001*
	Female	2501 (70%)	816 (46%)	
Estimated Glomerular Filtration Rate Stage	Rate < 90 ml/min.:	2440 (68%)	1134 (64%)	P = 0.0007*
	Rate ≥ 90 ml/min.:	1133 (32%)	649 (36%)	
Family history of hypertension	Yes	1276 (36%)	702 (39%)	P = 0.0097*
Family history of CVD	Yes	1247 (35%)	760 (43%)	P < 0.0001*
Smoking Status (Daily)	Never	1650 (46%)	827 (46%)	P = 0.0001*
	Formerly	878 (25%)	516 (29%)	
	Currently	1045 (29%)	440 (25%)	
Socioeconomic Status:	Secondary school	698 (20%)	471 (26%)	P < 0.0001*

**Feature**	**Levels**	Individuals with normal BP or lower, n=3 573	Individuals with high normal BP, n=1 783	t / χ^2, P value [a]
Highest level of education attained	Upper secondary school	1378 (39%)	698 (39%)	
	High school	432 (12%)	188 (11%)	
	Higher education, < 4 years	646 (18%)	262 (15%)	
	Higher education, ≥ 4 years	419 (12%)	164 (9%)	
Physical Activity, measured in Physical Activity Intelligence (PAI).	Low (< 50 PAI)	1213 (34%)	605 (34%)	P = 0.5389
	Medium (50-99 PAI)	921 (26%)	437 (25%)	
	High (≥ 100 PAI)	1439 (40%)	741 (42%)	
Marital status	Never married	925 (26%)	437 (25%)	P = 0.2113
	Married	2386 (67%)	1231 (69%)	
	Divorced	262 (7%)	115 (6%)	
Hypertension outcome	Yes	578 (16%)	756 (42%)	P < 0.0001*

Reported as mean (standard deviation) for numerical features, and count (percentage) for categorical features. P values were rounded to four decimals.

* Significant after applying Holm's step-down correction with α = 0.05.

[a] Two-tailed Welch t-test for a difference in mean value for numerical features or chi-square test for a difference in proportions in categorical features. Each individual feature was tested as the group of individuals having normal BP (<130/85 mmHg) versus the group of individuals having high normal BP (130/85 mmHg ≤ BP < 140/90 mmHg) at baseline.

'BMI': Body Mass Index, 'CVD': Cardiovascular disease.

Supplementary Table S8. Feature and outcome distributions of records in the test set stratified by individuals having optimal BP (<120/80 mmHg) or higher than optimal BP (120/80 mmHg ≤ BP < 140/90 mmHg) at baseline.

**Feature**	**Levels**	Individuals with optimal BP, n=1 809	Individuals with higher than optimal BP, n=3 547	t / χ^2, p value [a]
Age (years)	-	40.12 (10.64)	43.73 (12.01)	P < 0.0001*
Height (cm)	-	168.56 (7.79)	171.76 (8.99)	P < 0.0001*
BMI (m/kg^2)	-	24.53 (3.52)	25.79 (3.6)	P < 0.0001*
Systolic blood pressure (mmHg)	-	111.71 (5.64)	128.7 (5.96)	P < 0.0001*
Diastolic blood pressure (mmHg)	-	68.35 (6.11)	76.86 (6.84)	P < 0.0001*
Serum Creatinine (µmol/L)	-	63.29 (12.23)	68.45 (12.76)	P < 0.0001*
Serum Cholesterol (mmol/L)	-	5.34 (1.07)	5.71 (1.15)	P < 0.0001*
Serum high-density lipid Cholesterol (mmol/L)	-	1.47 (0.36)	1.4 (0.38)	P < 0.0001*
Serum Triglyceride (mmol/L)	-	1.29 (0.72)	1.6 (0.99)	P < 0.0001*
Non-fasting Serum Glucose (mmol/L)	-	4.96 (0.82)	5.17 (0.87)	P < 0.0001*
Sex	Male	365 (20%)	1674 (47%)	P < 0.0001*
	Female	1444 (80%)	1873 (53%)	
Estimated Glomerular Filtration Rate Stage	Rate < 90 ml/min.:	1247 (69%)	2327 (66%)	P = 0.0158
	Rate ≥ 90 ml/min.:	562 (31%)	1220 (34%)	
Family history of hypertension	Yes	629 (35%)	1349 (38%)	P = 0.0209
Family history of CVD	Yes	588 (33%)	1419 (40%)	P < 0.0001*
	Never	832 (46%)	1645 (46%)	P = 0.0018*

**Feature**	**Levels**	Individuals with optimal BP, n=1 809	Individuals with higher than optimal BP, n=3 547	t / χ^2, p value [a]
Smoking Status (Daily)	Formerly	428 (24%)	966 (27%)	
	Currently	549 (30%)	936 (26%)	
Socioeconomic Status: Highest level of education attained	Secondary school	309 (17%)	860 (24%)	P < 0.0001*
	Upper secondary school	699 (39%)	1377 (39%)	
	High school	230 (13%)	390 (11%)	
	Higher education, < 4 years	340 (19%)	568 (16%)	
	Higher education, ≥ 4 years	231 (13%)	352 (10%)	
Physical Activity, measured in Physical Activity Intelligence (PAI).	Low (< 50 PAI)	612 (34%)	1206 (34%)	P = 0.8848
	Medium (50-99 PAI)	466 (26%)	892 (25%)	
	High (≥ 100 PAI)	731 (40%)	1449 (41%)	
Marital status	Never married	473 (26%)	889 (25%)	P = 0.1615
	Married	1195 (66%)	2422 (68%)	
	Divorced	141 (8%)	236 (7%)	
Hypertension outcome	Yes	180 (10%)	1154 (33%)	P < 0.0001*

Reported as mean (standard deviation) for numerical features, and count (percentage) for categorical features. P values were rounded to four decimals.

* Significant after applying Holm's step-down correction with α = 0.05.

[a] Two-tailed Welch t-test for a difference in mean value for numerical features or chi-square test for a difference in proportions in categorical features. Each individual feature was tested as the group of individuals having optimal BP (<120/80 mmHg) versus the group of individuals with higher than optimal BP (120/80 mmHg ≤ BP < 140/90 mmHg) at baseline.

'BMI': Body Mass Index, 'CVD': Cardiovascular disease.

Supplementary Table S9. Feature and outcome distributions of the full data set including records with missing entries, and the complete data set without missing entries.

**Feature**	**Levels**	Full dataset, n=22 939	Complete dataset, n=17 852	t / χ^2, p value [a]	**Missing from full dataset (%)**
Age (years)	-	42.93 (12.03)	42.53 (11.68)	P = 0.0003*	-
Height (cm)	-	170.65 (8.8)	170.59 (8.74)	P = 0.2465	16 (< 0.1 %)
BMI (m/kg^2)	-	25.42 (3.65)	25.38 (3.63)	P = 0.1158	49 (0.2%)
Systolic blood pressure (mmHg)	-	122.96 (9.97)	122.85 (9.96)	P = 0.1416	-
Diastolic blood pressure (mmHg)	-	73.91 (7.75)	73.83 (7.76)	P = 0.1354	-
Serum Creatinine (µmol/L)	-	66.82 (13.02)	66.67 (12.96)	P = 0.1131	-
Serum Cholesterol (mmol/L)	-	5.59 (1.14)	5.56 (1.13)	P = 0.0043	-
Serum high-density lipid Cholesterol (mmol/L)	-	1.42 (0.38)	1.42 (0.38)	P = 0.0222	6 (< 0.1%)
Serum Triglyceride (mmol/L)	-	1.51 (0.94)	1.49 (0.93)	P = 0.0082	-
Non-fasting Serum Glucose (mmol/L)	-	5.12 (0.85)	5.1 (0.84)	P = 0.0309	-
Sex	Male	9 017 (39%)	6 748 (38%)	P = 0.0020*	-
	Female	13 922 (61%)	11 104 (62%)		
Estimated Glomerular Filtration Rate Stage	Rate ≤ 90 ml/min.:	15 278 (67%)	11 890 (67%)	P = 0.9994	-
	Rate ≥ 90 ml/min.:	7 659 (33%)	5 962 (33%)		

**Feature**	**Levels**	Full dataset, n=22 939	Complete dataset, n=17 852	t / χ^2, p value [a]	**Missing from full dataset (%)**
Family history of hypertension	Yes	6 980 (30%)	6 432 (36%)	P = 0.5157	3388 (14.8 %)
Family history of CVD	Yes	8 344 (36%)	6 730 (38%)	P = 0.7410	903 (3.9 %)
Smoking Status (Daily)	Never	10 294 (45%)	8 237 (46%)	P = 0.0106	189 (0.8 %)
	Formerly	5 855 (26%)	4 678 (26%)		
	Currently	6 601 (29%)	4 937 (28%)		
Socioeconomic Status: Highest level of education attained	Secondary school	5 338 (23%)	3 843 (22%)	P < 0.0001*	321 (1.4 %)
	Upper secondary school	8 816 (38%)	6 969 (39%)		
	High school	2 558 (11%)	2 063 (12%)		
	Higher education, < 4 years	3 608 (16%)	3 036 (17%)		
	Higher education, ≥ 4 years	2 298 (10%)	1 941 (11%)		
Physical Activity, measured in Physical Activity Intelligence (PAI).	Low (< 50 PAI)	7 686 (34%)	6 088 (34%)	P = 0.2621	889 (3.9 %)
	Medium (50-99 PAI)	5 809 (25%)	4 726 (26%)		
	High (≥ 100 PAI)	8 555 (37%)	7 038 (39%)		
Marital status	Never married	5 786 (25%)	4 453 (25%)	P = 0.5461	59 (0.3 %)
	Married	15 427 (67%)	12 127 (68%)		
	Divorced	1 667 (7%)	1 272 (7%)		
Hypertension at follow-up	Yes	5 736 (25%)	4 358 (24%)	P = 0.1717	-

Reported as mean (standard deviation) for numerical features, and count (percentage) for categorical features. P values were rounded to four decimals.

* Significant after applying Holm's step-down correction with α = 0.05.

[a] Two-tailed Welch t-test for a difference in mean value for numerical features or chi-square test for a difference in proportions in categorical features. Each individual feature was tested

**Feature**	**Levels**	Full dataset, n=22 939	Complete dataset, n=17 852	t / χ^2, p value [a]	**Missing from full dataset (%)**

as the group of individuals from the full dataset versus the group in the complete dataset. 'BMI': Body Mass Index, 'CVD': Cardiovascular disease.

Supplementary Table S10. Performance on the test set for models fitted with the training data, with and without records that were imputed using MICE for both data sets.

**Models, training data**	**AUC (↑)**		**Scaled Brier (↑)**		**ICI (↓)**	
	Test data, imputed, n=6 883	Test data, n=5 356	Test data, imputed, n=6 883	Test data, n=5 356	Test data, imputed, n=6 883	Test data, n=5 356
XGBoost						
- Imputed, n=16 056	0.829	0.794	0.264	0.202	0.022	0.014
- Not imputed, n=12 496	0.813	0.795	0.235	0.204	0.017	0.016
Elastic regression						
- Imputed, n=16 056	0.799	0.795	0.209	0.204	0.02	0.017
- Not imputed, n=12 496	0.799	0.795	0.208	0.204	0.02	0.016
KNN						
- Imputed, n=16 056	0.801	0.783	0.211	0.185	0.017	0.014
- Not imputed, n=12 496	0.799	0.786	0.198	0.186	0.037	0.024
Logistic regression						
- Imputed, n=16 056	0.782	0.78	0.185	0.181	0.015	0.015
- Not imputed, n=12 496	0.782	0.78	0.185	0.181	0.014	0.014
High normal BP rule*						
-	0.657	0.656	-	-	-	-
Framingham risk model						
-	0.791	0.786	0.09	0.078	0.114	0.115
Framingham risk model, recalibrated						
- Imputed, n=16 056	0.791	0.786	0.201	0.192	0.11	0.01
- Not imputed, n=12 496	0.791	0.786	0.201	0.192	0.011	0.01

Performance obtained for models fitted with, and without, imputed training data applied on imputed and non-imputed test data. Reported as mean after bootstrapping. The symbols (↑) and (↓) signify increasing or decreasing values as improved performance, respectively. Results from the main analysis can be seen in the bottom right square of each quadrant, as the

intersection of “- Not imputed” training data and “Test data” for each model.
* Scaled Brier score and ICI is omitted for ‘High normal BP rule’ as calibration is not meaningful when all predictions are either 0% or 100% risk.

Supplementary Table S11. Feature distributions of the complete data set used in the main analysis compared with the complete data set derived from those lost to follow-up.

**Feature**	**Levels**	**Included, complete data, n=17 852**	**Lost to follow-up, complete data, n = 8050**	t / χ^2, P value [a]
Age (years)	-	42.53 (11.68)	39.9 (16.15)	P < 0.0001*
Height (cm)	-	170.59 (8.74)	171.1 (9.01)	P < 0.0001*
BMI (m/kg^2)	-	25.38 (3.63)	25.37 (4)	P = 0.4678
Systolic blood pressure (mmHg)	-	122.85 (9.96)	123.33 (10.01)	P = 0.0002*
Diastolic blood pressure (mmHg)	-	73.83 (7.76)	73.01 (8.02)	P < 0.0001*
Serum Creatinine (µmol/L)	-	66.67 (12.96)	67.64 (14.44)	P < 0.0001*
Serum Cholesterol (mmol/L)	-	5.56 (1.13)	5.44 (1.19)	P < 0.0001*
Serum high-density lipid Cholesterol (mmol/L)	-	1.42 (0.38)	1.37 (0.37)	P < 0.0001*
Serum Triglyceride (mmol/L)	-	1.49 (0.93)	1.53 (0.95)	P = 0.0028*
Non-fasting Serum Glucose (mmol/L)	-	5.1 (0.84)	5.1 (0.86)	P = 0.4562
Sex	Male	6 748 (38%)	3516 (44%)	P < 0.0001*
	Female	11 104 (62%)	4534 (56%)	
Estimated Glomerular Filtration Rate Stage	Rate < 90 ml/min.:	11 890 (67%)	5745 (71%)	P < 0.0001*
	Rate ≥ 90 ml/min.:	5 962 (33%)	2305 (29%)	

Feature	Levels	Included, complete data, n=17 852	Lost to follow-up, complete data, n = 8050	t / χ^2, P value [a]
Family history of hypertension	Yes	6 432 (36%)	2527 (31%)	P < 0.0001*
Family history of CVD	Yes	6 730 (38%)	2555 (32%)	P < 0.0001*
Smoking Status (Daily)	Never	8 237 (46%)	3337 (41%)	P < 0.0001*
	Formerly	4 678 (26%)	1644 (20%)	
	Currently	4 937 (28%)	3069 (38%)	
Socioeconomic Status: Highest level of education attained	Secondary school	3 843 (22%)	1857 (23%)	P < 0.0001*
	Upper secondary school	6 969 (39%)	2757 (34%)	
	High school	2 063 (12%)	1328 (16%)	
	Higher education, < 4 years	3 036 (17%)	1339 (17%)	
	Higher education, ≥ 4 years	1 941 (11%)	769 (10%)	
Physical Activity, measured in Physical Activity Intelligence (PAI).	Low (< 50 PAI)	6 088 (34%)	2855 (35%)	P = 0.0089*
	Medium (50-99 PAI)	4 726 (26%)	1993 (25%)	
	High (≥ 100 PAI)	7 038 (39%)	3202 (40%)	
Marital status	Never married	4 453 (25%)	3536 (44%)	P < 0.0001*
	Married	12 127 (68%)	3847 (48%)	
	Divorced	1 272 (7%)	667 (8%)	

Reported as mean (standard deviation) for numerical features, and count (percentage) for categorical features. P values were rounded to four decimals.

* Significant after applying Holm's step-down correction with α = 0.05.

[a] Two-tailed Welch t-test for a difference in mean value for numerical features or chi-square test for a difference in proportions in categorical features. Each individual feature was tested as the group of individuals from the full dataset versus the group in the complete dataset.

'BMI': Body Mass Index, 'CVD': Cardiovascular disease.

References

1. Moons, K. G. M. et al. Risk prediction models: II. External validation, model updating, and impact assessment. Heart **98**, 691 (2012).
2. Parikh, N. I. et al. A risk score for predicting near-term incidence of hypertension: The Framingham Heart Study. Annals of Internal Medicine vol. 148 102–110 (2008).
3. Chien, K.-L. et al. Prediction models for the risk of new-onset hypertension in ethnic Chinese in Taiwan. J Hum Hypertens **25**, 294–303 (2011).
4. Lim, N.-K., Lee, J.-W. & Park, H.-Y. Validation of the Korean Genome Epidemiology Study Risk Score to Predict Incident Hypertension in a Large Nationwide Korean Cohort. Circ J **80**, 1578–1582 (2016).
5. Koohi, F. et al. Validation of the Framingham hypertension risk score in a middle eastern population: Tehran lipid and glucose study (TLGS). BMC PUBLIC HEALTH **21**, (2021).
6. Namgung, H. K. et al. Development and validation of hypertension prediction models: The Korean Genome and Epidemiology Study_Cardiovascular Disease Association Study (KoGES_CAVAS). J Hum Hypertens (2022) doi:10.1038/s41371-021-00645-x.
7. Moons, K. G. M. et al. PROBAST: A Tool to Assess Risk of Bias and Applicability of Prediction Model Studies: Explanation and Elaboration. Ann Intern Med **170**, W1 (2019).
